# Use of human splenocytes in an innovative humanised mouse model for prediction of immunotherapy‐induced cytokine release syndrome

**DOI:** 10.1002/cti2.1202

**Published:** 2020-11-04

**Authors:** Alba Matas‐Céspedes, Lee Brown, Krishnaa T Mahbubani, Bethany Bareham, Jackie Higgins, Michelle Curran, Lolke de Haan, Jean‐Martin Lapointe, Richard Stebbings, Kourosh Saeb‐Parsy

**Affiliations:** ^1^ Clinical Pharmacology and Safety Sciences R&D AstraZeneca Cambridge UK; ^2^ Department of Surgery University of Cambridge and NIHR Cambridge Biomedical Campus Cambridge UK; ^3^ Present address: ADC Therapeutics London UK

**Keywords:** animal models, cytokine release syndrome, humanized mice, immune system, immunotherapy, pre‐clinical safety assessment

## Abstract

**Objectives:**

Humanised mice have emerged as valuable models for pre‐clinical testing of the safety and efficacy of immunotherapies. Given the variety of models available, selection of the most appropriate humanised mouse model is critical in study design. Here, we aimed to develop a model for predicting cytokine release syndrome (CRS) while minimising graft‐*versus*‐host disease (GvHD).

**Methods:**

To overcome donor‐induced variation, we directly compared the *in vitro* and *in vivo* immune phenotype of immunodeficient NSG mice reconstituted with human bone marrow (BM) CD34^+^ haematopoietic stem cells (HSCs), peripheral blood mononuclear cells (PBMCs) or spleen mononuclear cells (SPMCs) from the same human donors. SPMC engraftment in NSG‐dKO mice, which lack MHC class I and II, was also evaluated as a strategy to limit GvHD. Another group of mice was engrafted with umbilical cord blood (UCB) CD34^+^ HSCs. Induction of CRS *in vivo* was investigated upon administration of the anti‐CD3 monoclonal antibody OKT3.

**Results:**

PBMC‐ and SPMC‐reconstituted NSG mice showed short‐term survival, with engrafted human T cells exhibiting mostly an effector memory phenotype. Survival in SPMC‐reconstituted NSG‐dKO mice was significantly longer. Conversely, both BM and UCB‐HSC models showed longer survival, without demonstrable GvHD and a more naïve T‐cell phenotype. PBMC‐ and SPMC‐reconstituted mice, but not BM‐HSC or UCB‐HSC mice, experienced severe clinical signs of CRS upon administration of OKT3.

**Conclusion:**

PBMC‐ and SPMC‐reconstituted NSG mice better predict OKT3‐mediated CRS. The SPMC model allows generation of large experimental groups, and the use of NSG‐dKO mice mitigates the limitation of early GvHD.

## Graphical abstract

In this study, we perform a head‐to‐head comparison between different humanised mouse models in terms of survival, human immune engraftment and response to immunotherapy. We characterise the differences in the immune compartment of these models and demonstrate that some, but not all, can mimic the human immune response to immunotherapies.

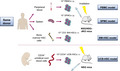

## Introduction

Great advances have been made in the development of new therapeutic modalities that target the human immune system in different scenarios.^1^, ^2^ However, these immunotherapies can lead to severe and undesired adverse effects, and effective pre‐clinical animal models are essential for safety and efficacy evaluation before transition into clinical trials. Traditionally, toxicology studies rely on the evaluation of human biologics in non‐human primates and of surrogates in rodents.^3^ However, due to evolutionary divergence, animal models may not always predict toxicity in humans and could lead to the induction of cytokine release syndrome (CRS). CRS is a life‐threatening systemic inflammatory response, resulting from a massive release of cytokines from targeted and bystander immune cells, which in extreme cases can lead to multiorgan failure.^4^, ^5^, ^6^ An unfortunate example is TGN1412, an anti‐CD28 superagonistic monoclonal antibody (mAb). Pre‐clinical testing of TGN1412 *in vitro*, in rodents with a surrogate and in cynomolgus monkeys with the drug candidate, was well tolerated with no sign of CRS. Nonetheless, in the first‐in‐human trial unexpected severe toxicity occurred, with development of CRS and lymphopenia,^6^ which was subsequently attributed to inter‐species differences in the expression of CD28 on CD4^+^ effector memory T cells.^7^, ^8^ Similarly, muromonab (OKT3), a murine anti‐CD3 mAb that was developed to treat acute rejection after organ transplantation, led to CRS in patients, with increased plasma levels of TNF‐α, IFN‐γ and IL‐2, detected 1–2 h after administration. Moreover, at this time point lymphocyte counts in blood circulation had dropped in treated patients, and lymphopenia persisted for more than 24 h.^9^, ^10^

Immunodeficient mice reconstituted with human immune cells, or humanised mice, are emerging as valuable *in vivo* models to investigate human immune responses. Several different humanised mouse models have recently been developed as pre‐clinical models in translational research.^11^, ^12^, ^13^, ^14^, ^15^ The most common background strain of immunodeficient mice used to achieve efficient levels of humanisation is NOD‐*scid IL2*Rγ^null^ (NSG). These mice lack murine T, B and natural killer (NK) cells, have defective dendritic cells and macrophages, and impaired innate immunity.^16^, ^17^

The reconstitution of immunodeficient mice with mature human peripheral blood mononuclear cells (PBMCs) is one of the easiest methods of humanisation and was first described by Mosier *et al*. in 1988.^18^ This model allows the examination of the human immune response without delay in engraftment, as the human leukocytes transferred into the mice are already mature. Human T cells with a memory and activated phenotype are predominantly present in these humanised mice.^11^, ^12^ However, the main disadvantage of this model is the early development of graft‐*versus*‐host disease (GvHD) at four to eight weeks post‐engraftment, most likely triggered by the recognition of murine antigens by the human lymphocytes. This xenogenic immune response effectively reduces the experimental window, so that only short‐term experiments are feasible.^19^ Longer experimental windows may be possible with a new strain of NSG mice deficient in the murine major histocompatibility complex (MHC) class I and II (NSG‐dKO mice), which show increased survival after PBMC humanisation and delayed GvHD.^20^

Alternatively, immunodeficient mice can be reconstituted with human haematopoietic stem cells (HSCs), obtained from either umbilical cord blood (UCB),^21^ bone marrow (BM),^22^, ^23^ foetal liver^23^ or cytokine‐mobilised from the BM into the peripheral blood.^24^ HSC‐reconstitution of humanised mice leads to the generation of multiple lineages of human immune cells and survival of mice for more than 20 weeks. The main caveats of these models are the time taken to *de novo* generate human immune cells and achieve stable human engraftment in mice, together with the lack of a fully functional human immune system, as most of the T, B, NK cells and monocytes engrafted display some impaired responses to antigens.^25^, ^26^, ^27^ To overcome this disadvantage, transgenic mouse strains that express human cytokines or specific HLA types have been developed.^28^, ^29^, ^30^, ^31^, ^32^

Taken together, there are various humanised mouse models that potentially could better predict human immunotherapy toxicities. Several studies have recently used these models to study CRS induction after immunotherapy treatment.^33^, ^34^, ^35^, ^36^, ^37^ However, each of these models has a number of limitations that first need to be better understood. The biological variation between human donors and the limited per‐donor availability of immune cells can lead to small experimental groups, which is a major cofounder in many studies utilising humanised mouse models. In this study, we characterise and compare head‐to‐head the human immune engraftment and survival of humanised mice generated by reconstitution with either human PBMCs, spleen mononuclear cells (SPMCs), UCB‐derived CD34^+^ HSCs or BM‐derived CD34^+^ HSCs, to elucidate the advantages and limitations of each model to predict CRS. To avoid donor‐related variation, we used PBMCs, SPMCs and BM‐HSCs from the same donors for human immune reconstitution in mice. We demonstrate, for the first time, the potential of SPMCs derived from deceased human organ donors for the generation of large numbers of humanised mice and that GvHD can be ameliorated by reconstitution of NSG‐dKO mice with SPMCs. We further show that the PBMC and SPMC models, but not BM‐HSC and UCB‐HSC models, develop CRS upon treatment with OKT3 *in vivo*.

## Results

### Survival and immune reconstitution patterns in the different humanised mouse models

**Figure 1:**
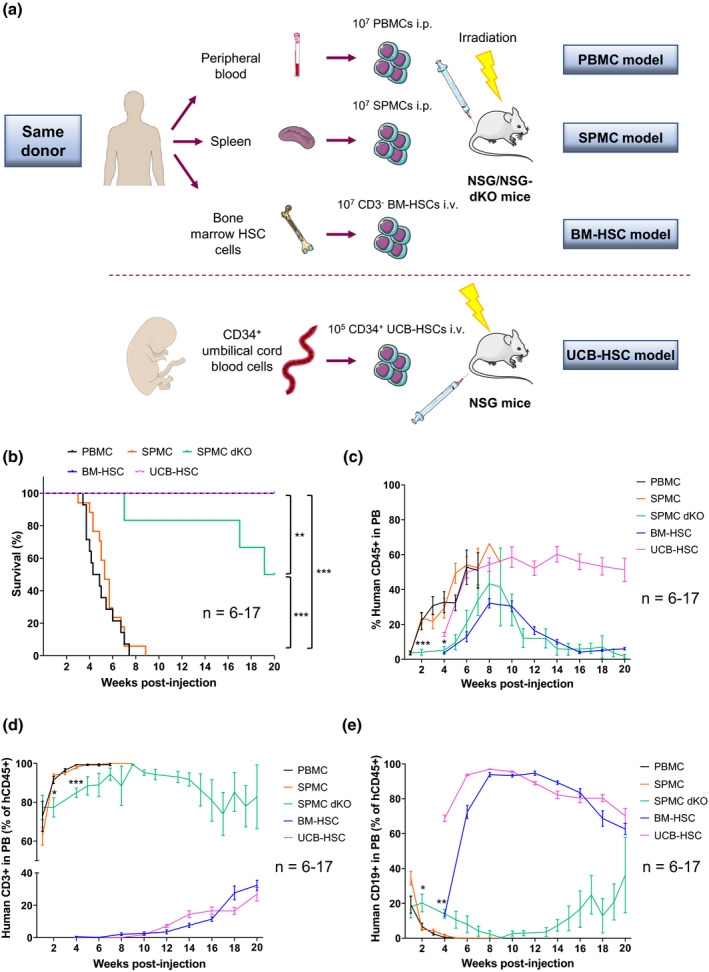
Distinct survival and human reconstitution in the different humanised mouse models. **(a)** Schematic representation of the generation of different humanised mouse models. To prevent donor‐related variations, PBMCs, SPMCs and BM‐HSCs used for reconstitution of NSG mice, were isolated from the same three adult donors (Donors 1–3). For the SPMC dKO model, NSG‐dKO mice were humanised with SPMCs from Donor 3. For the UCB‐HSC model, NSG mice were reconstituted with UCB‐HSCs cells from Donor 4. **(b)** Kaplan–Meier plot showing overall survival of the different humanised mouse models. Mice were sacrificed at 20 weeks or earlier if they showed clinical signs of GvHD. **(c)** Levels of hCD45^+^ cells circulating in PB of mice at the indicated time points. **(d)** Levels of hCD3^+^ T cells and **(e)** hCD19^+^ B cells circulating in PB of mice. Data in the PBMC (*n* = 14), SPMC (*n* = 17) and BM‐HSC (*n* = 13) models are representative of three independent experiments with Donors 1–3 in each model. Data in the SPMC dKO model (*n* = 6) are representative of one experiment with Donor 3. Data in the UCB‐HSC (*n* = 7) are representative of one experiment with Donor 4. Data are mean ± SEM. The Mantel–Cox test was used to assess statistical differences between models in **b**, and a two‐way ANOVA to assess differences at each time point between models in **c–e**; *P < 0.05, **P < 0.01, ***P < 0.001. BM‐HSC, bone marrow haematopoietic stem cells; i.p., intraperitoneally; i.v., intravenously; NSG, NOD scid gamma; NSG‐dKO, NOD scid gamma double knockout; PB, peripheral blood; PBMC, peripheral blood mononuclear cells; SPMC dKO, SPMC model in NSG‐dKO mice; SPMC, spleen mononuclear cells; UCB‐HSC, umbilical cord blood haematopoietic stem cells.

We compared the survival and human immune phenotype of humanised mice generated by reconstitution of irradiated immunodeficient NSG or NSG‐dKO mice, with either human PBMCs, SPMCs, BM‐HSCs or UCB‐HSCs, as represented in Figure 1a. The donor demographics are shown in Supplementary table 1.

NSG mice in the PBMC and SPMC models had a similar short survival and needed to be sacrificed between week 3 and 9 after immune reconstitution, due to early development of GvHD (Figure 1b). In comparison, survival of NSG‐dKO^20^ mice reconstituted with SPMCs from the same donor was significantly extended, with five out of six mice alive 16 weeks after reconstitution and three mice surviving to study end (week 20) without clinical signs of GvHD (Figure 1b). In contrast, all NSG mice in the BM‐HSC and UCB‐HSC models survived long term, without signs of ill‐health until the end of the study at week 20 (Figure 1b).

Low levels of hCD45^+^ cells circulating in mouse blood were detected one week after human immune reconstitution in the PBMC and SPMC models (< 3%), and increased steadily until the experiment endpoint, which indicated human leukocyte expansion in mice (Figure 1c). Of note, the human subpopulations of hCD45^+^ cells circulating in blood were predominantly (> 90%) hCD3^+^ T cells (Figure 1d), with minimal hCD19^+^ B cells (Figure 1e) and hCD14^+^ monocytes (Supplementary figure 1) from the second week post‐engraftment. We observed significantly slower engraftment kinetics in SPMC‐reconstituted NSG‐dKO mice compared to NSG mice, with low levels of hCD45^+^ cells in blood during weeks 1 to 4 (< 6%), which peaked after 9 weeks (~41%) and then decreased to stable levels of 5–12% until the experiment endpoint (Figure 1c). Importantly, expansion of hCD3^+^ T cells was significantly retarded in NSG‐dKO mice, at levels of ~80% during the first 4 weeks (Figure 1d), accompanied by significant higher levels of hCD19^+^ B cells (15‐20%) compared to NSG mice during the same time (Figure 1e).

Levels of hCD45^+^ cells in the BM‐HSC model increased from week 4, peaked at weeks 8–10 and then decreased to stable levels of 6–10% until the experiment endpoint (Figure 1c). In the UCB‐HSC model, *de novo* generated hCD45^+^ were found at low levels in circulating blood 4 weeks after reconstitution, followed by a rapid increase to a plateau from week 8–10 onwards (~55%) (Figure 1c). hCD19^+^ B cells were the predominant (> 70%) subpopulation of hCD45^+^ cells in blood in the BM‐HSC and UCB‐HSC models from week 4 (Figure 1e). In contrast, hCD3^+^ T cells were only detected from week 10‐12 (Figure 1d). hCD3^+^ T‐cell levels continued to increase steadily over time, reaching constant levels of 15–20% within hCD45 population by weeks 18–20. Importantly, hCD14^+^ monocytes were found circulating in mouse blood at low levels, 1–2% of hCD45^+^ throughout the experiment (Supplementary figure 1), supporting the multilineage haematopoietic development of HSC models^38^.

### Differential infiltration of mouse organs with human cells across models

On flow cytometric assessment, the spleen was the most efficiently repopulated lymphoid tissue across all models, containing a higher proportion of human cells than in circulating blood. Accordingly, hCD45^+^ cell levels in the spleen of PBMC‐ and SPMC‐reconstituted NSG mice were > 80% of total hCD45^+^cells, whereas this was approximately 35% in SPMC engrafted NSG‐dKO mice and in BM‐HSC mice, and 58% in UCB‐HSC mice (Supplementary figure 2). In addition, human cell engraftment in the bone marrow of mice was greater in BM‐HSC and UCB‐HSC models than in PBMC and SPMC models (~28% and 58% vs ~13% and 20%, respectively), demonstrating a niche‐like migration of human HSC cells (Supplementary figure 2).

**Figure 2:**
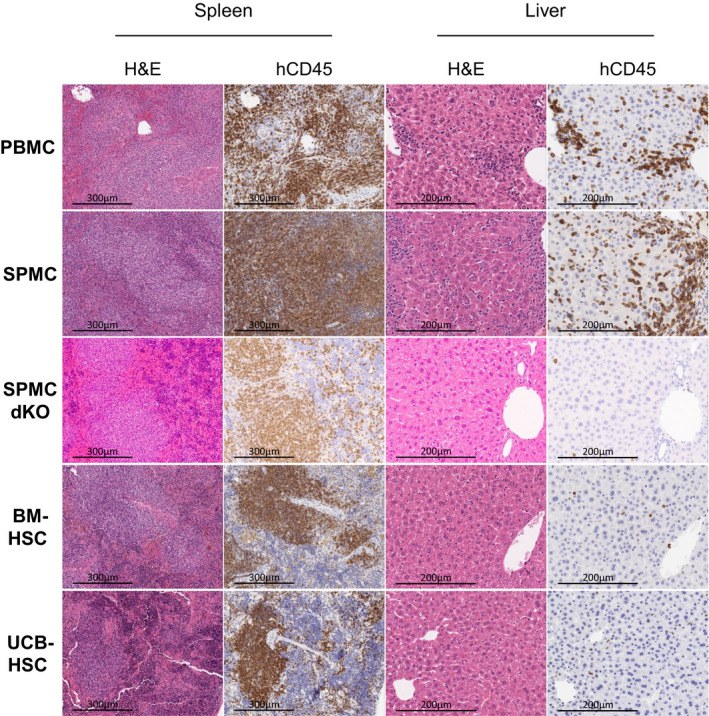
Histological comparison of human immune cell infiltration and inflammation levels in mouse tissue. Representative images of the H&E and hCD45 staining on the spleen and liver of one mouse in each model, reconstituted with Donor 3 (PBMC, SPMC, SPMC dKO and BM‐HSC models) and Donor 4 (UCB‐HSC model). PBMC and SPMC mice shown were sacrificed at week 5 and 6, after GvHD development. SPMC dKO, BM‐HSC and UCB‐HSC mice shown were sacrificed at study end at week 20. Magnification: ×400 for the liver and ×200 for the spleen. BM‐HSC, bone marrow haematopoietic stem cells; H&E, haematoxylin and eosin; PBMC, peripheral blood mononuclear cells; SPMC dKO, SPMC model in NSG double knockout mice; SPMC, spleen mononuclear cells; UCB‐HSC, umbilical cord blood haematopoietic stem cells.

PBMC‐ and SPMC‐reconstituted NSG mice showed substantial immune cell infiltration of all tissues examined on immunohistochemistry (IHC) (Figure 2 and Supplementary figure 3). The spleen was the most extensively infiltrated organ, consistent with flow cytometry data. There was expansion of the splenic white pulp periarteriolar lymphoid sheath areas, infiltrated with hCD45^+^ cells. The liver showed changes indicative of hepatitis, with infiltration of hCD45^+^ immune cells around centrolobular veins and in the portal areas and periportal lobular parenchyma, associated with necrosis of single hepatocytes. The percentage of hCD45^+^ area in this organ was ~5–7% (Figure 2). There was also considerable infiltration of hCD45^+^ immune cells in the lungs (~19–23% hCD45^+^ area), with cells accumulating around blood vessels and airways, and extending into the alveolar interstitium (Supplementary figure 3). The kidneys also showed infiltration by hCD45^+^ cells (~2–5% hCD45^+^ area), predominantly under the pelvic epithelium and around cortical blood vessels (Supplementary figure 3). These findings are considered consistent with manifestations of GvHD, mediated by hCD45^+^ immune cells. The histological patterns and the overall severity of the of immune cell infiltration were similar between the PBMC and SPMC models.

In contrast, SPMC‐reconstituted NSG‐dKO mice that survived to the study endpoint (20 weeks), and NSG mice in the BM‐HSC and UCB‐HSC models, only showed histologically visible changes in the spleen, with a mild degree of splenic white pulp expansion by hCD45^+^ cells (Figure 2). Small numbers of hCD45^+^ cells (< 1% hCD45^+^ area) were scattered throughout the liver, lung and kidney, but without substantial aggregation or evidence of GvHD‐type inflammatory lesions (Figure 2 and Supplementary figure 3).

### Phenotypic characterisation of engrafted human leucocytes in humanised mouse models

**Figure 3:**
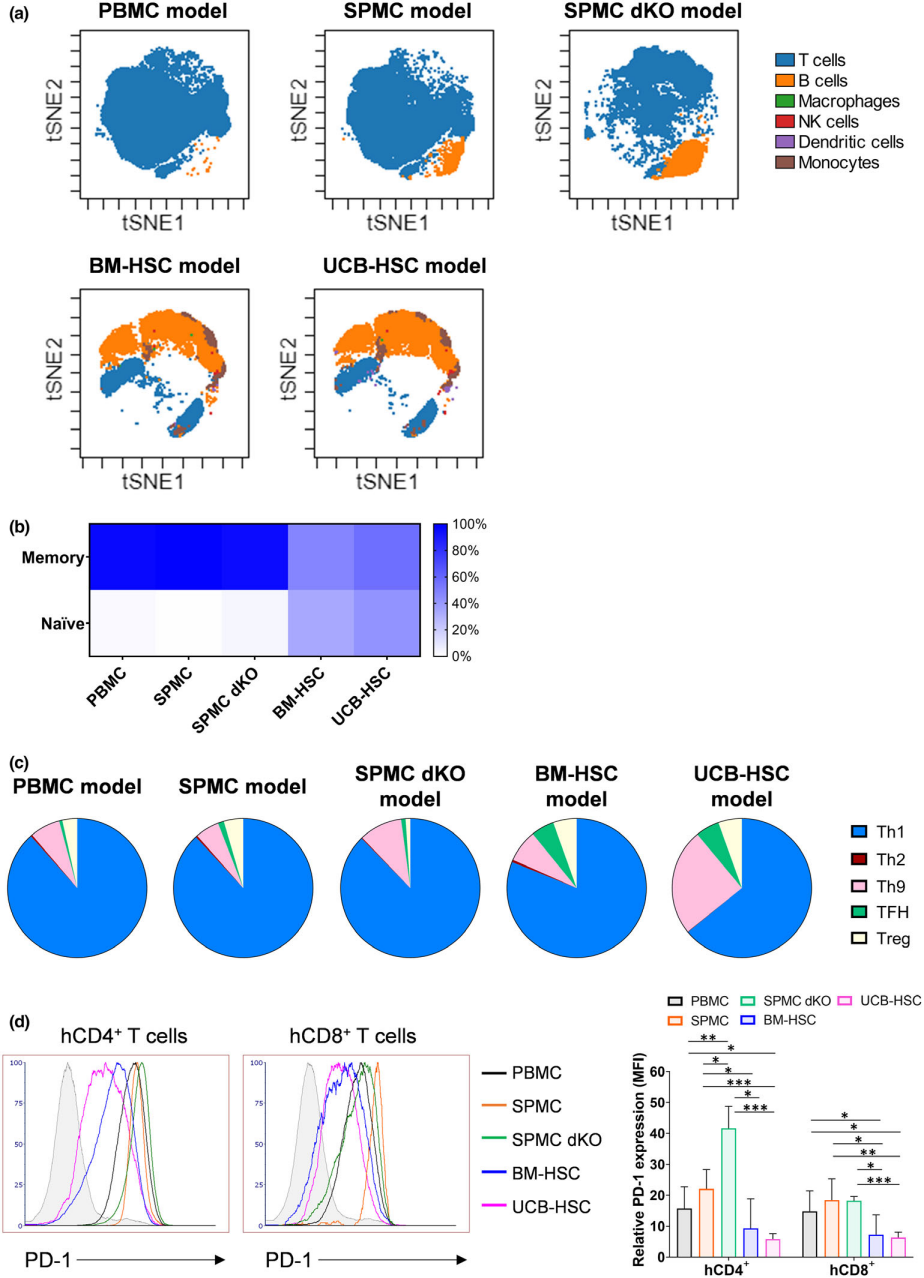
Immunophenotyping of hCD45^+^ cells infiltrating the spleen of the different humanised mouse models. **(a)** Visualisation using t‐SNE (t‐stochastic neighbour embedding) of immune subpopulations within hCD45^+^ cells infiltrating the spleen of one representative mouse in each model (Donors 3 and 4) at sacrifice analysed by flow cytometry. **(b)** Percentage of memory phenotype on hCD3^+^ T cells infiltrating the spleen of mice. **(c)** Representative data of hCD4^+^ T helper cell subtypes and their proportions of the whole hCD4^+^ population. Mean of all donors combined is shown in **b** and **c**. **(d)** Expression of PD‐1 on hCD4^+^ and hCD8^+^ T cells. Histograms are representative of one mouse in each model (Donors 3 and 4). Relative PD‐1 expression on hCD4^+^ and hCD8^+^ T cells in the spleen of mice. The MFI is plotted as a relative fold difference as compared to the FMO controls. Mean ± SD are shown. Mice and donor numbers are the same as in Figure 1. A two‐way ANOVA was used to determine significant differences between models; *P < 0.05, **P < 0.01, ***P < 0.001. BM‐HSC, bone marrow haematopoietic stem cells; FMO, fluorescence minus one; MFI, mean fluorescence intensity; NK, natural killer; PBMC, peripheral blood mononuclear cells; SPMC dKO, SPMC model in NSG double knockout mice; SPMC, spleen mononuclear cells; TFH, T follicular helper; UCB‐HSC, umbilical cord blood haematopoietic stem cells.

Immunophenotyping of hCD45^+^ cells engrafted in mice, revealed a prominent infiltration of hCD3^+^ T cells (> 85%) in the spleen of PBMC‐ and SPMC‐reconstituted NSG or NSG‐dKO mice (Figure 3a). Fewer hCD19^+^ B cells were present in the PBMC model (~2%) than in the SPMC model with NSG mice (~8%), or with NSG‐dKO mice (~13%) (Figure 3a and Supplementary figure 4a). As expected^12^, engraftment of other human cell lineages in the spleen of mice, including monocytes, macrophages, NKs and dendritic cells was substantially lower (<0.6%) in these models (Figure 3a and Supplementary figure 4a), although they were a component of the initial PBMCs and SPMCs inoculums (Supplementary figure 5a, b). In contrast, BM‐HSC and UCB‐HSC models showed a diverse repertoire of human immune populations engrafting the spleen at sacrifice (Figure 3a). A significantly higher proportion of hCD19^+^ B cells was found in these models (BM‐HSC ~ 49%; UCB‐HSC ~ 76%), together with a significantly lower percentage of hCD3^+^ T cells (BM‐HSC ~ 48%; UCB‐HSC ~ 20%) and higher levels of myeloid cells (Figure 3a and Supplementary figure 4a).

The majority (> 90%) of hCD3^+^ T cells in the PBMC‐ and SPMC‐reconstituted NSG or NSG‐dKO mice exhibited a memory phenotype (Figure 3b), suggesting xeno‐activation of transferred hCD3^+^ T cells from the initial inoculums (Supplementary figure 5c). In comparison, a significantly lower percentage of hCD3^+^ memory cells was found in the BM‐HSC and UCB‐HSC models (47–56%), along with a higher proportion of hCD3^+^ naïve cells (34–42%) (Figure 3b). The hCD4:hCD8 ratio in the spleen of PBMC and SPMC NSG mice was ~1:1.5 (Supplementary figure 4b), similar to that in the original human donor samples (Supplementary figure 5d). In contrast, in SPMC‐reconstituted NSG‐dKO mice the ratio was ~4:1 (Supplementary figure 4b), demonstrating an increased expansion of hCD4^+^ cells in these mice. A hCD4:hCD8 ratio of ~1:1.2 was detected in BM‐HSC mice and of ~1.2:1 in UCB‐HSC mice (Supplementary figure 4b). hCD4^+^ T cells in these latter models showed a significantly lower percentage of Th1 cells (< 45%), and a more diverse range of hCD4^+^ subpopulations than in the PBMC‐ and SPMC‐reconstituted NSG or NSG‐dKO mice, where the majority (> 90%) of hCD4^+^ T cells showed a Th1 phenotype (Figure 3c and Supplementary figure 4c). The same pattern was observed within hCD8^+^ T cells among all models (Supplementary figure 4d). Interestingly, hCD4^+^ Tregs were found at levels of 3‐5% in all mouse models (Supplementary figure 4c), which was similar to the levels present in the original donor PBMC and SPMC samples (2–3%) (Supplementary figure 5e). The majority of hCD4^+^ and hCD8^+^ cells expressed the activation marker PD‐1 in the PBMC‐ and SPMC‐reconstituted NSG or NSG‐dKO mice, but the expression was significantly lower in the BM‐HSC and UCB‐HSC models (Figure 3d).

At termination, the immunophenotype of human cells circulating in blood and infiltrating the spleen of humanised mice across models was comparable, with similar distribution of hCD45^+^ subpopulations (Supplementary figure 6a) and human T‐cell immunophenotype (Supplementary figure 6b–g).

### Impaired human CD3^+^ T‐cell function *in vitro* after engraftment in mice

**Figure 4:**
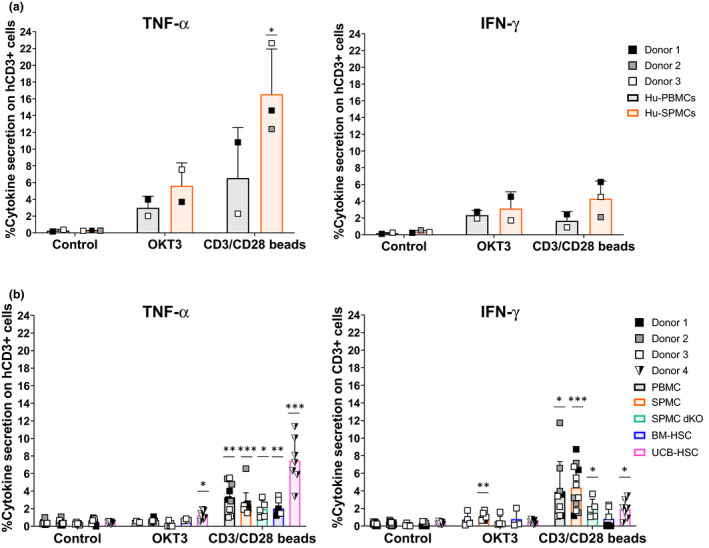
Intracellular cytokine production by human T cells pre‐ and post‐engraftment in mice. **(a)** Intracellular cytokine production by Hu‐PBMCs (*n* = 2) and Hu‐SPMCs (*n* = 3) samples before engraftment into mice, after 24 h of culture *in vitro*. The percentage of TNF‐α (left panel) and IFN‐γ (right panel) positive hCD3^+^ T cells is shown. Data from three independent experiments with Donors 1–3 are represented. **(b)** Intracellular cytokine production by human lymphocytes isolated from the spleen of humanised mice at the experiment endpoint, after 24 h of culture *in vitro*. For the PBMC (*n* = 4–9), SPMC (*n* = 6–12) and the BM‐HSC (*n* = 3–7) models, data from three independent experiments with Donors 1–3 in each model are shown. In the SPMC dKO model (*n* = 3–5) data are representative of one experiment with Donor 3. In the UCB‐HSC model (*n* = 7) data are representative of one experiment with Donor 4. Individual data and mean ± SD of all donors combined are shown. The non‐paired *t*‐test and two‐way ANOVA were used to determine significant differences between control and treated groups in each model; *P < 0.05, **P < 0.01, ***P < 0.001. BM‐HSC, bone marrow haematopoietic stem cells; Hu‐PBMCs, human PBMCs inoculum; Hu‐SPMCs, human SPMCs inoculum; PBMC, peripheral blood mononuclear cells; SPMC, spleen mononuclear cells; SPMC dKO, SPMC model in NSG double knockout mice; UCB‐HSC, umbilical cord blood haematopoietic stem cells.

Activation and cytokine release by hCD3^+^ T cells is thought to be critical for CRS induction. To determine the functionality of hCD3^+^ T cells engrafted in the spleen of different humanised mouse models, we first evaluated their ability *in vitro* to produce cytokines, accompanied by activation and proliferation. Similar to pre‐engraftment human PBMCs and SPMCs inoculums (Figure 4a), hCD3^+^ T cells isolated from the spleen of humanised mice produced intracellular TNF‐α in all models after co‐culture with CD3/CD28 beads (2.5–7.5%) (Figure 4b, left). A significant increase in intracellular IFN‐γ was also observed in the PBMC, SPMC and UCB‐HSC models after this stimulus (Figure 4b, right). In contrast, stimulation with OKT3 induced only low levels of cytokine production on hCD3^+^ T cells post‐engraftment in mice, with a modest increase in TNF‐α only in the UCB‐HSC model and of IFN‐γ in the SPMC engrafted NSG mice (Figure 4b). These results demonstrate that hCD3^+^ T cells derived from the spleen of humanised mice can still induce cytokine secretion *in vitro*, but mainly after strong stimulation with the CD3/CD28 beads. Furthermore, we observed a slightly increased IFN‐γ production rather than TNF‐α in the hCD3^+^ T cells derived from PBMC and SPMC engrafted mice, while a greater TNF‐α production was detected in the original PBMCs and SPMCs (Figure 4a).

**Figure 5:**
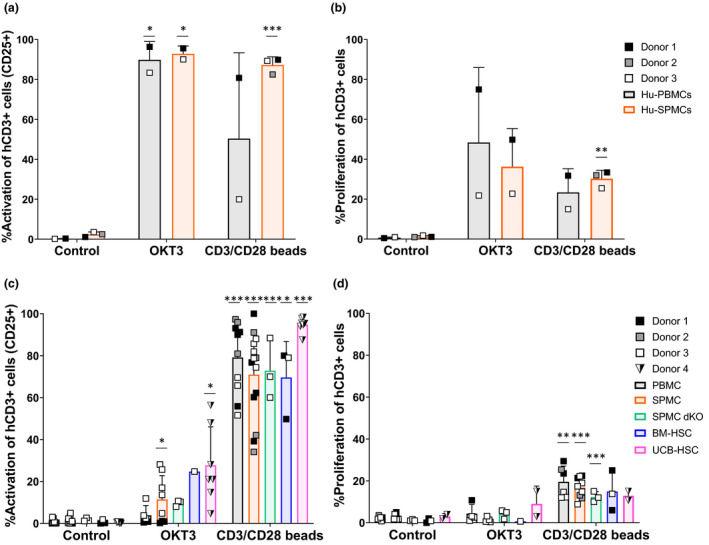
Activation and proliferation of human T cells pre‐ and post‐engraftment in mice. **(a)** Percentage of activated hCD3^+^ T cells (CD25^+^) and **(b)** proliferating hCD3^+^ T cells (CFSE^Low^) in Hu‐PBMCs and Hu‐SPMCs samples before engraftment into mice, after 72 h of *in vitro* culture. Data of three independent experiments with Donors 1–3 are shown. **(c)** Percentage of activated hCD3^+^ T cells (CD25^+^) and **(d)** proliferating hCD3^+^ T cells (CFSE^Low^) isolated from the spleen of humanised mice at the experiment endpoint, after 72 h of *in vitro* culture. Mice and donor numbers are the same as in Figure 4b. Individual data and mean ± SD of all donors combined are shown. The non‐paired *t*‐test and two‐way ANOVA were used to determine significant differences between control and treated groups in each model; *P < 0.05, **P < 0.01, ***P < 0.001. BM‐HSC, bone marrow haematopoietic stem cells; Hu‐PBMCs, human PBMCs inoculum; Hu‐SPMCs, human SPMCs inoculum; PBMC, peripheral blood mononuclear cells; SPMC dKO, SPMC model in NSG double knockout mice; SPMC, spleen mononuclear cells; UCB‐HSC, umbilical cord blood haematopoietic stem cells.

Similar to the activation and proliferation levels of human PBMCs and SPMCs inoculums (Figure 5a, b), > 70% of hCD3^+^ T cells isolated from mouse spleen showed significant levels of CD25 expression, indicative of an activated phenotype, after incubation with CD3/CD28 beads (Figure 5c). This activation was accompanied by a substantial increase in proliferating hCD3^+^ T cells (> 17%) in the PBMC and SPMC models compared to their controls (Figure 5d). Similar to the cytokine release, stimulation with OKT3 resulted in less activation of hCD3^+^ T cells (Figure 5c), together with reduced proliferation levels in all models (Figure 5d).

### Only PBMC and SPMC humanised mice recapitulate OKT3‐induced cytokine release syndrome (CRS)

**Figure 6:**
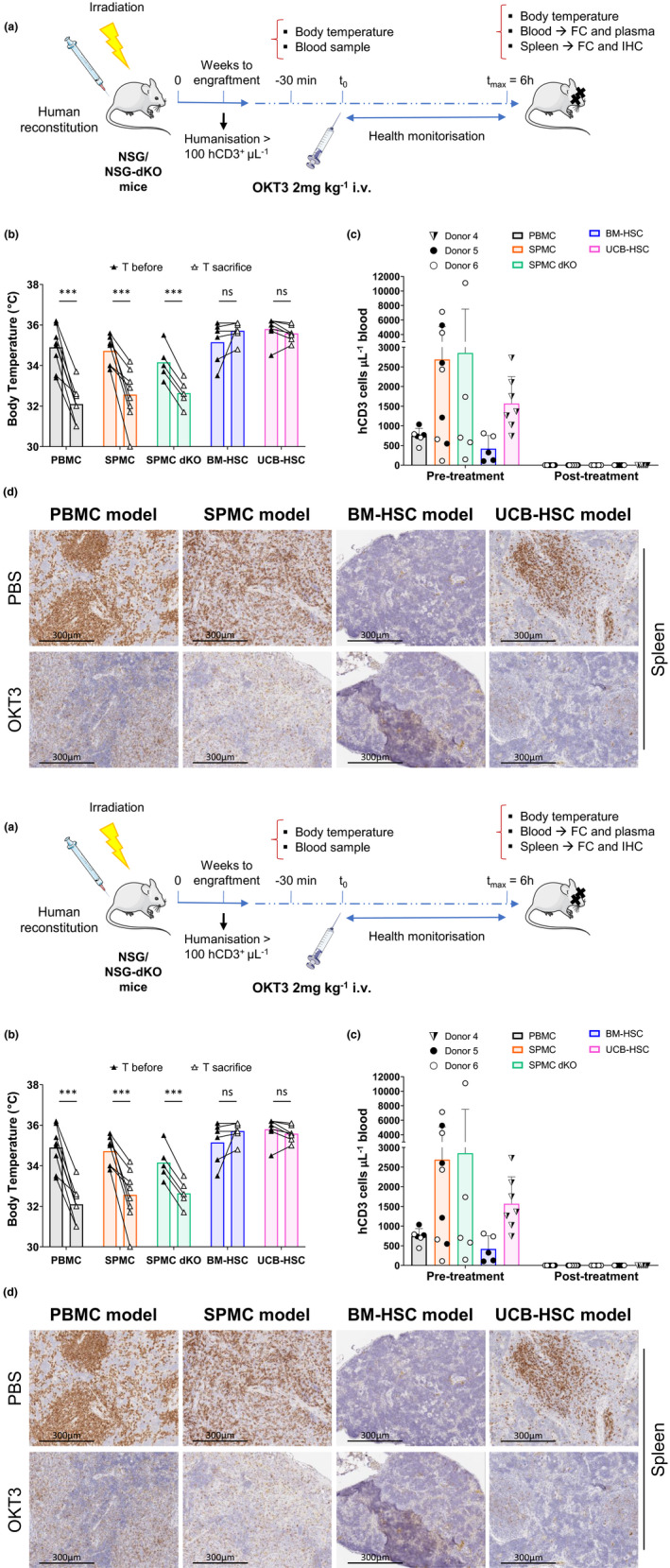
Response to OKT3 treatment in different humanised mouse models. **(a)** Experimental design to evaluate induction of cytokine release syndrome in humanised mice. **(b)** Body temperature (°C) of OKT3‐treated mice before and 6 h after treatment. The paired *t‐*test was used to determine significant differences before‐after OKT3 treatment. **(c)** Number of hCD3^+^ T cells per μL of mouse blood assessed before and 6 h after OKT3 treatment. **(d)** Representative images of the hCD3^+^ T‐cell staining of the spleen of one PBS and one OKT3‐treated mouse in each model, corresponding to mice reconstituted with Donors 4 (UCB‐HSC model) and 5 (PBMC, SPMC and BM‐HSC models), magnification ×200. **(e)** Human cytokines secreted into mouse plasma evaluated in PBS and OKT3 treated mice at sacrifice. For the PBMC (*n* = 6 or 7), SPMC (*n* = 8 or 9) and the BM‐HSC (*n* = 5 or 6) models, data from two independent experiments with Donors 5 and 6 in each model are shown. Data in the NSG‐dKO mice (*n* = 3–5) correspond to one experiment reconstituting with SPMCs cells from Donor 6. In the UCB‐HSC model (*n* = 6 or 7) data are representative of two independent experiments with Donor 4. Individual mouse data and mean ± SD of all donors combined are shown. The non‐paired *t‐*test was used to determine significant differences between PBS and OKT3 treated mice in each model; *P < 0.05, **P < 0.01, ***P < 0.001. BM‐HSC, bone marrow haematopoietic stem cells; FC, flow cytometry; IHC, immunohistochemistry; NSG‐dKO, NOD scid gamma double knock out; PBMC, peripheral blood mononuclear cells; SPMC dKO, SPMC model in NSG double knockout mice; SPMC, spleen mononuclear cells; T, temperature; UCB‐HSC, umbilical cord blood haematopoietic stem cells.

We next compared the effect of i.v. OKT3 administration and the induction of CRS in the different humanised mouse models generated using cells from the same donors, as detailed in Figure 6a. NSG mice in the PBMC and SPMC models and SPMC engrafted NSG‐dKO mice rapidly exhibited clinical signs of ill‐health after OKT3 treatment, showing impaired mobility, hunched posture, piloerection and a drop in body temperature detectable as soon as 1h post injection and progressively worsening until the time of sacrifice at 6h (T ~ 32°C). In contrast, OKT3‐treated mice in the BM‐HSC and UCB‐HSC models did not display any adverse signs and their temperature remained stable (T ~ 35.5°C) (Figure 6b). Nevertheless, treatment with OKT3 induced lymphopenia, as evidenced by a marked reduction in hCD45^+^ peripheral blood cells in all humanised mouse models (Supplementary figure 8a), with a selective hCD3^+^ T‐cell loss from peripheral blood and a complete downregulation of hCD3^+^ expression (Figure 6c), as reported in patients.^9^, ^10^ Furthermore, fewer hCD4^+^ and hCD8^+^ T cells remained in circulating blood in all models after OKT3 treatment, but with downregulated human TCR/CD3 complex (Supplementary figure 8b–f), as previously reported in humans.^39^, ^40^

We also detected by IHC, reduced expression of hCD3^+^ T cells in spleen and lungs of OKT3‐treated mice, compared to the group administered with PBS (Figure 6d and Supplementary figure 9a), indicating that OKT3 had efficiently reached these organs. Following further analysis of the spleen by flow cytometry, we corroborated a complete downregulation of the human TCR/CD3 complex in OKT3‐treated groups, while the percentage of hCD4^+^ and hCD8^+^ T cells remained unchanged compared to controls (Supplementary figure 10a‐e). These data confirmed that hCD3^+^ T cells were not depleted by OKT3, but rather the human TCR/CD3 complex was downregulated, consistent with the mode of action of OKT3.^39^, ^40^

Finally, a different pattern of cytokine secretion was uncovered in plasma across humanised mouse models upon OKT3 treatment. As shown in Figure 6e, higher levels of human IL10, TNF and IFN‐γ were released in PBMC, SPMC and SPMC‐NSG‐dKO mice compared to BM‐HSC and UCB‐HSC mice. In contrast, IL‐2 was similarly secreted in PBMC, SPMC, SPMC dKO and UCB‐HSC mice. OKT3 also induced some release of IL‐4, IL‐6 and IL‐17A, but to considerably lower absolute levels in all models (Supplementary figure 9b).

## Discussion

Humanised mice are increasingly recognised as promising models in translational research permitting the investigation of human immune responses in different scenarios.^14^, ^38^, ^41^ However, due to the great variety of humanised mouse models available, the choice of the most appropriate model should be made in accordance with the research question to address. In the present work, we report for the first time a detailed comparison of the different humanised mouse models generated by reconstitution with human cells from the same donors, to reduce donor‐related variations.

Our results represent a first proof of concept that effective humanisation can be achieved after reconstitution of mice with SPMCs isolated from the spleen of human organ donors. Much higher numbers of lymphocytes (~1 × 10^10–11^; data not shown) can be isolated from a human spleen compared to a peripheral blood sample. Therefore, it is possible to generate substantially larger groups of humanised mice from SPMCs, thus enabling experiments to be repeated using the same donor material. Of note, the lymphocyte distribution in the human spleen is different to that in the peripheral blood, containing more B cells than T cells and a greater number of activated T cells with memory phenotype (Supplementary figure 5a–c), in agreement with the important role of the spleen in the immune cell homeostasis and induction of systemic immune responses.^42^, ^43^ Despite these phenotypic differences, we observed a similarly short survival in NSG mice reconstituted with either SPMCs or PBMCs, due to early development of GvHD. In both models, a predominant human T‐cell expansion was observed over time, with an activated memory phenotype constituting mostly Th1 and Tc1 subtypes. Although other human cell subpopulations were present in the initial PBMC and SPMC inoculums, the major expansion of T cells in the murine environment and the lack of human specific cytokines needed, might impair their survival.^44^, ^45^ Nonetheless, the observed higher number of hCD19^+^ B cells infiltrating the spleen of mice in the SPMC model compared to the PBMC model could be attributed to the higher percentage of hCD19^+^ B cells in the initial SPMCs inoculum, which are likely to express chemokine receptors for homing to the spleen of mice. Overall, the repopulation capacity of human cells was comparable across PBMC and SPMC models, with human leucocytes present in all mouse organs examined and with inflammation of similar severity.

A major limitation that arises in the PBMC and SPMC models is the early development of GvHD, which reduces the available experimental window. This xenogeneic reaction is thought to be mainly due to the recognition of the murine MHC antigens by human T cells, inducing their subsequent activation and expansion.^19^ Hence, to overcome this short‐term survival, we engrafted human SPMCs into NSG‐dKO mice, which lack murine MHC class I and II.^20^ The overall survival was significantly extended in most of NSG‐dKO mice beyond 16 weeks, in comparison to NSG mice engrafted with SPMCs from the same donor, which developed GvHD within 7 weeks. Human CD45^+^ cells were found in circulating blood of NSG‐dKO mice one week after humanisation, but with a significantly slower T‐cell expansion kinetics. This is consistent with a reduced xeno‐activation of human T cells that results in a lower expansion and reactivity to the mouse environment. Interestingly, hCD3^+^ T cells in NSG‐dKO mice at sacrifice had a T memory phenotype, with high expression of the activation marker PD‐1, as previously reported by Brehm *et al*.^20^ This suggests that despite the lack of murine MHC class I and II molecules, which are the major targets of the xeno‐response, there are other xeno‐antigens that activate human T cells in these mouse models and can lead to GvHD development over time.^20^ Nevertheless, this activation was slower than in NSG mice, allowing the favorable expansion of hCD4^+^ T cells with better tolerance to the host. The number of hCD8^+^ T cells, which are the most potent effectors mediating GvHD,^46^ was also lower than in NSG mice. The reduced T‐cell expansion was corroborated by a lower infiltration of hCD45^+^ cells in solid organs in NSG‐dKO mice and the absence of inflammatory lesions in those mice that survived until the study end.

In contrast, HSC reconstituted mice experienced a *de novo* generation of multiple human cell populations differentiated in mouse environment, with a stable human repopulation from week 18 onwards. We observed significantly greater generation of human leucocytes in mice reconstituted with HSC cells derived from UCB than in mice reconstituted with adult BM‐HSCs, in accordance with a major pluripotent capacity retained in UCB‐HSCs than in adult HSCs.^23^, ^47^ Due to this more persistent and robust human engraftment, UCB samples are commonly the source of choice by many research groups for HSC transplantation in mice. Despite the differences in engraftment, the infiltration of human leucocytes in mouse tissues was comparable between BM‐HSC and UCB‐HSC models, with T cells, B cells, NK cells and myeloid cells in similar proportions. These mice survived for more than 20 weeks without development of GvHD. The absence of GvHD can be explained by the negative selection that human HSCs undergo during differentiation in the mouse environment, making these human cells more tolerant to mouse‐derived antigens and with higher activation thresholds^26^, ^27^.

The *in vitro* evaluation of human T‐cell activity in humanised mice demonstrated an impaired hCD3^+^ T‐cell function in all models and the need for a strong stimulation to be responsive, in comparison to the cytokine production and proliferation levels achieved on human T cells pre‐engraftment. In the HSC models, this impairment of T‐cell function *in vitro* had been previously reported^25^, ^26^ and could be attributed to an anergic state of these cells in mice, due to the limited education of human T cells during their haematopoiesis on mouse MHC molecules, together with the lack of human cytokines needed to develop a fully functional human immune system. Conversely, in the PBMC and SPMC models in NSG mice, the rapid activation and expansion of human T cells in response to the xeno‐reactive mouse environment, could lead to T‐cell exhaustion over time. This phenomenon may be the consequence of a protracted T‐cell response, where T‐cell stimulation continues as the antigen cannot be cleared.^48^ In these models, stimulation of mouse‐derived hCD3^+^ T cells *in vitro* with the anti‐CD3 mAb OKT3, was not sufficient to result in cytokine production, activation and proliferation of hCD3^+^ T cells. In contrast, incubation with CD3/CD28 beads, which provide coupled stimulatory signals, achieved cytokine production, activation and proliferation of hCD3^+^ T cells post‐engraftment, but to a lower extent than that observed with the original PBMCs and SPMCs samples. In addition, the shift in the cytokine pattern towards IFN‐γ production rather than TNF‐α post‐engraftment is consistent with T‐cell xeno‐activation and exhaustion, as this cytokine has a role in T‐cell homeostasis limiting the expansion of T cells especially after prolonged antigen stimulation.^48^, ^49^, ^50^, ^51^ The *in vitro* functionality of hCD3^+^ T cells derived from NSG‐dKO mice was similar to NSG mice, with cells reacting and achieving cytokine production, activation and proliferation only after strong CD3/CD28 stimulation. Again, this relative anergy is likely due to the prolonged exposure to the murine environment, as NSG‐dKO mice had a significant longer survival than NSG after SPMCs reconstitution. Collectively, these data suggest that the human T cells isolated from humanised mice require strong stimulation *in vitro* to be rescued from their anergic state.

The *in vivo* comparison of CRS induction after i.v. administration of OKT3 revealed different responses across the humanised mouse models. PBMC‐ and SPMC‐reconstituted NSG mice, together with SPMC NSG‐dKO mice, manifested severe adverse clinical signs and a significant drop in body temperature early after treatment, consistent with previous reports using PBMC‐reconstituted humanised mice.^33^, ^34^, ^37^ However, BM‐HSC and UCB‐HSC mice did not exhibit any symptoms of illness and their temperature remained constant for the 6 hours of experiment. Interestingly, all models demonstrated a marked lymphopenia, with a significant loss of hCD3^+^ T cells from circulation, as observed in patients.^9^, ^10^ Furthermore, we found some remaining levels of hCD4^+^ and hCD8^+^ T cells in circulation that did not express either hCD3 nor TCRα/β, suggesting that most human T cells were cleared from peripheral blood but those remaining had downregulated TCR/CD3 complex. This mechanism of action has been described for CD3‐specific antibodies after binding CD3, resulting in the internalisation or capping of TCR/CD3 complex from cell surface.^40^ This phenomenon was also observed in spleen and lungs from OKT3‐treated mice. We did not find major changes in the proportion of hCD4^+^ or hCD8^+^ T cells infiltrating the spleen in OKT3‐treated groups, compared to their controls in any mouse model. This finding was also reported by Weißmüller *et al*.^34^ in lymph nodes, thymus and peritoneum of humanised mice, suggesting that human T cells cleared from circulating blood did not migrate to solid organs after treatment.

We detected a broad range of human cytokines secreted in OKT3‐treated mice in all models compared to their controls, but a considerably higher secretion in PBMC, SPMC and SPMC‐NSG‐dKO mice. The pro‐inflammatory cytokines known to be highly released upon administration of OKT3 in patients, TNF‐α and IL‐2, were detected at lower concentrations in these models, probably due to the reduced number of human cells in these mice compared to humans.^9^, ^10^ Nonetheless, IFN‐γ levels were greatly increased in these humanised mouse models,^52^ which may be a consequence of the shift in the cytokine pattern described above. Of note, the total number of hCD3^+^ T cells circulating in mouse blood on the day of the experiment was different across models, as depicted in Figure 6c. However, these differences did not correlate with a more severe response to OKT3 and increased secretion of cytokines. As demonstrated in the PBMC model, although the average number of hCD3^+^ T cells in circulation was lower than in the UCB‐HSC model, all the OKT3 treated mice in the PBMC model suffered severe clinical signs accompanied by an extensive secretion of human cytokines. In addition, the intra‐group variations on hCD3^+^ T‐cell numbers did not correlate with increases in adverse effects in mice. Hence, the distinct response to OKT3 may be attributed to the different levels of maturation of hCD3^+^ T cells across models. Our data are in accordance with previous reports where OKT3 showed induction of cytokine release on activated hCD3^+^ T cells but not on naïve T cells.^53^ Hence, suggesting that the limited education of hCD3^+^ T cells in the BM‐HSC and UCB‐HSC models^44^, ^54^ impaired their response to OKT3 *in vivo*.

Interestingly, fever is one of the most common symptoms of CRS in patients, due to the systemic release of pro‐inflammatory cytokines.^4^, ^5^, ^9^, ^10^ In contrast, PBMC, SPMC and SPMC‐NSG‐dKO reconstituted mice, experienced a significant drop in body temperature after OKT3 administration, despite secreting human pro‐inflammatory cytokines. The mechanism underlying this effect is not clear yet, and has also been reported by others using these models to evaluate therapeutic‐related CRS.^33^, ^34^, ^37^ A possible explanation is cytokine species‐specificity, as the human cytokines secreted are likely to have differential effects in these mice, with their cognate human receptors potentially only expressed on engrafted cells, and signalling of human cytokine through mouse cytokine receptors probably limited.^55^ Thus, human IFN‐γ which is highly secreted in PBMC and SPMC models, does not cross‐react with mouse receptors.^55^, ^56^ In contrast, human TNF‐α is potentially cross‐reactive as it shares 78% homology with its mouse counterpart.^19^, ^55^ Moreover, both NSG and NSG‐dKO mice have a complete null mutation of the gamma chain of the IL‐2 receptor (*IL2*Rγ^null^),^16^ which is a common component of the cell surface receptors of IL‐2, IL‐4, IL‐7, IL‐9, IL‐15 and IL‐21. Hence, although some of these human cytokines are cross‐reactive in mice, their signalling pathways are blocked. However, other cytokines released by human T cells upon OKT3 treatment, could also be involved in the hypothermia and contribute to the rapid development of symptoms in PBMC, SPMC and SPMC‐NSG‐dKO humanised mouse models. Despite these inter‐species differences, the information derived from the toxicity studies in the aforementioned models, demonstrate that these models recapitulated the side effect of OKT3 treatment in patients, including lymphopenia, downregulation of the hTCR/CD3 complex and CRS, secreting a wide range of cytokines. Moreover, our data confirmed a similar *in vivo* response to OKT3 between SPMC‐reconstituted NSG and NSG‐dKO mice, with a significant longer survival observed in NSG‐dKO mice, allowing their experimental use for a longer duration after humanisation.

Humanised mice have been used by others to evaluate CRS upon mAb treatment. In a recent study, Ye *et al*.^37^ demonstrated the efficacy of using PBMC‐reconstituted NSG, NSG‐dKO or NSG‐SGM3 (expressing hIL‐3, GM‐CSF and SCF) mice to evaluate the induction of CRS after OKT3, TGN1412 analogue or Keytruda (anti‐PD‐1) treatment. These models showed a rapid and strong response, capturing the variation in CRS between different donors and in a drug‐dependent manner, reproducing the response observed in patients. Similarly, Weißmüller *et al*.^34^ reported severe adverse reactions and CRS induction after OKT3 or TGN1412 treatment of PBMC‐engrafted NRG or NRG HLA‐DQ8 + Ab1^null^ transgenic mice. No differences in response between non‐transgenic and DQ transgenic mice were detected, suggesting no benefit for an HLA‐DQ transgenic mouse in the PBMC model. The use of a more sophisticated model, the NOG‐BLT (bone marrow‐liver‐thymus) model, where mice develop a fully engrafted human immune system has also been explored,^35^, ^36^ with the advantage that these mice could be more responsive to a larger diversity of immunotherapies due to the greater diversity of immune populations present. However, the limited availability of human foetal tissues is a major challenge in the development of this model.

Collectively, our findings suggest that humanised mouse models have the potential to be valuable models for pre‐clinical safety testing of immunotherapies and can mimic several of the severe adverse effects induced upon mAb treatment in patients. However, careful selection of the humanised mouse model is crucial, as we have demonstrated that the distinct models are incumbered by different limitations. These models continue to evolve, adding more complexity and overcoming deficiencies. Hence, as sophistication raises, their utility will also likely grow. An increasing interest in using these models for efficacy evaluation of immune‐oncology drugs has also arisen in the last years. In this context, the potential to establish primary tumors in humanised mice reconstituted with a human immune system, including from autologous patients, represents an attractive opportunity in the immunotherapy field.

## Methods

### Human samples

BM, peripheral blood and spleen samples were obtained from deceased transplant organ donors after obtaining informed consent from the donor families with full ethical approval (15/EE/0152, NRES Committee East of England – Cambridge South). Peripheral blood samples were taken before the cessation of circulation. Spleen and bone marrow samples were taken immediately after the removal of organs for transplantation. BM was aspirated from the thoracic and/or lumbar vertebrae of deceased donors. Human UCB‐derived CD34^+^ HSCs, positively isolated via immunomagnetic separation with a purity > 90%, were purchased from Lonza (Basel, Switzerland). Donor characteristics are summarised in Supplementary table 1.

For the human donor spleen, a single cell suspension was prepared in RPMI‐1640 (Gibco, Paisley, UK) supplemented with 10% FBS (Gibco), following mechanical tissue dissociation with the gentleMACS^TM^ dissociator (Miltenyi Biotec, Bergisch Gladbach, Germany) and filtration of the cellular fraction through a 70‐µm cell strainer (Corning, NY, USA). For the human donor BM, the aspirated sample was diluted in RPMI‐1640 + 10% FBS and filtered through a 70‐µm cell strainer. Human donor peripheral blood was diluted in RPMI‐1640 + 10% FBS medium. Mononuclear cells were isolated from the single cell suspension of the spleen, BM or peripheral blood of the donors by gradient centrifugation on Lymphoprep™ (StemCell Technologies, Vancouver, BC, Canada) at 800 *g*, for 20 min, at room temperature (RT). Erythrocytes were lysed with red blood cell lysis (RBCL) buffer when necessary. Mononuclear cells were cryopreserved in liquid nitrogen in 90% FBS + 10% DMSO and stored within the Department of Surgery for future use.

### Mice

NOD.Cg‐Prkdc^SCID^Il2rg^tm1Wjl^/SzJ (NSG) mice and NSG‐(*K^b^D^b^*)^null^ (*IA*)^null^ mice (referred to as NSG‐double knockout: NSG‐dKO), were purchased from Charles River (Saffron Walden, UK) and The Jackson Laboratory (Sacramento, CA, USA) respectively, and used to set up breeding colonies that were housed in individually ventilated cages and maintained in a pathogen‐free facility at the Central Biomedical Services, University of Cambridge. All experimental procedures were approved by the United Kingdom Home Office under the Animal (Scientific Procedures) Act 1986 (PPL 70/8702 and P57643EBB).

### Immune reconstitution

For the PBMC and SPMC models, 6–8 week‐old NSG or NSG‐dKO mice were weighed and irradiated at 2.3Gy using a Caesium‐137 source, 4–24 h before immune reconstitution. Human PBMCs or SPMCs were defrosted, resuspended in PBS + 2% FBS and 1 × 10^7^ cells injected into each mouse intraperitoneally (i.p.). Several SPMCs samples were incubated after defrosting with 0.04 mg mL^−1^ DNAse‐I (Sigma‐Aldrich, St. Louis, MO, USA) at 37°C for 30 min, to avoid clumping of cells.

For the BM‐HSC model, 5–6‐week‐old NSG females were weighed and irradiated at 2.5Gy using a Caesium‐137 source, 4–24 h before immune reconstitution. Human BM‐derived HSCs were defrosted and depleted for hCD3^+^ cells using anti‐CD3 coated microbeads (Milteny Biotec, Bergisch Gladbach, Germany), at a concentration recommended by the manufacturer, in the AutoMACS® Pro Separator (Milteny Biotec). The negative fraction was resuspended after depletion in PBS + 2% FBS, and 1 × 10^7^ cells injected into each mouse intravenously (i.v.).

For the UCB‐HSC model, 5–6‐week‐old NSG females were weighed and irradiated at 2.5Gy using a Caesium‐137 source, 4–24 h before the humanisation. Human UCB‐derived CD34^+^ HSCs were defrosted and resuspended in PBS + 2% FBS, and 1 × 10^5^ cells injected into each mouse i.v.

### Immune comparison study

To avoid donor‐related fluctuations (Figure 1a), irradiated NSG mice in the PBMC, SPMC and BM‐HSC models were reconstituted with human cells from the same adult donors (Donors 1–3) in three independent experiments (*n* = 4–6 per group). For the SPMC dKO model, irradiated NSG‐dKO mice (*n* = 6) were humanised with SPMCs from Donor 3. For the UCB‐HSC model, NSG mice (*n* = 7) were reconstituted with UCB‐HSCs cells obtained commercially (Donor 4).

Mice were monitored daily and were sacrificed if they lost > 15% of their body weight or exhibited signs of GvHD such as hunched posture, piloerection or reduced mobility. Humanisation levels in mice were monitored by flow cytometry either weekly or bi‐weekly, in tail blood samples collected in EDTA coated tubes. SPMC‐reconstituted NSG‐dKO mice that did not develop GvHD were sacrificed at 20 weeks. BM‐HSC and UCB‐HSC reconstituted mice were sacrificed after 20 weeks of human reconstitution, when they showed stable engraftment of human cells in blood as described previously^11^, ^16^.

### Flow cytometry

Tail blood samples were processed with RBCL buffer to lyse erythrocytes. Human immune cell populations were assessed using a FACSCanto II (BD Biosciences, San Jose, CA, USA) flow cytometer and stained with specific antibodies listed in Supplementary table 2. Data were analysed with FCS Express Research Edition (De Novo Software, Pasadena, CA, USA). Human cells were phenotyped as follows: leucocytes (hCD45^+^/mCD45.1^−^/7AAD^−^), T cells (hCD3^+^/hCD45^+^), B cells (hCD19^+^/hCD45^+^) and monocytes (hCD14^+^/hCD45^+^).

At sacrifice, single cell suspensions were prepared from spleen and BM of mice by mincing the spleen with the plunger of a syringe, or by flushing the tibiae and femurs with RPMI‐1640 and filtering through a 70‐µm cell strainer; whole blood was also collected in EDTA coated tubes. Erythrocytes were lysed and cells stained for immunophenotyping of human cells with specific antibodies listed in Supplementary table 2. Stained samples were fixed with 2% paraformaldehyde, then washed and run on a FACSymphony™ flow cytometer (BD Biosciences), acquiring at least 30000 hCD45^+^ events. Data were analysed with FCS Express Research Edition. Human cells were phenotyped as follows: leucocytes (hCD45^+^/mCD45.1^−^/FVD^−^), T cells (hCD3^+^/hCD45^+^), B cells (hCD19^+^/hCD45^+^), monocytes (hCD14^+^/hCD45^+^), macrophages (hCD206^+^/hCD45^+^) NK cells (hCD56^+^/hCD45^+^), Dendritic cells (hCD11c^+^/HLA‐DR^+^/hCD14^−^/hCD45^+^), T memory cells (hCD45RO^+^/hCD3^+^), naïve T cells (hCD45RA^+^/hCD3^+^), CD4^+^ T cells (hCD4^+^/hCD3^+^), Th1 (hCXCR3^+^/hCCR5^+^/hCD4^+^), Th2 (hCCR4^+^/hCCR6^−^/hCD4^+^), Th9 (hCCR4^−^/hCCR6^+^/hCD4^+^), T follicular helper (TFH) (hCXCR5^+^/hCD4^+^), Treg (hCD25^Hi^/hCD127^Low^/hCD4^+^), CD8^+^ T cells (hCD8^+^, hCD3^+^), Tc1 (hCXCR3^+^/hCCR5^+^/hCD8^+^) and Tc2 (hCCR4^+^/hCCR3^+^/hCD8^+^). Multidimensional data were represented using vi‐SNE and FlowSOM on the Cytobank platform (Beckman Coulter, Brea, CA, USA). vi‐SNE method was run processing 30 000 hCD45^+^ cells and setting the following parameters: iterations = 1000, perplexity = 30.

### Histology

All tissues were fixed in 10% neutral buffered formalin (Leica Biosystems, Wetzlar, Germany), processed and embedded into paraffin wax. Tissue sections (4 µm thick) were stained with haematoxylin & eosin (H&E). Additional serial sections were immunohistochemically stained for hCD45 (clone D9M8I, Cell Signaling Technology, Leiden, The Netherlands) and hCD3 (clone CD3‐12, Bio‐Rad, Watford, UK), using the automated staining platforms Leica Bond RX (Leica Biosystems) and Autostainer Link 48 (Dako Agilent, Santa Clara, CA, USA). The hCD45 IHC protocol had previously shown specificity for human cells, with absence of staining when applied to normal mouse lymphoid tissues (unpublished observations). Microscope slides were digitally scanned (Aperio, Leica Biosystems) to obtain whole slide scans. Sections stained with hCD45 were analysed for amount of positive staining using Halo image analysis software (Indica Labs, Albuquerque, NM, USA). Positive hCD45 staining was reported as % positive area over total tissue area.

### Cytokine production, activation and proliferation *in vitro*

Single cell suspensions from the spleen of humanised mice were prepared in RPMI‐1640 medium as described above. Human mononuclear cells were isolated by gradient centrifugation on Lymphoprep™ at 800 *g*, for 20 min, at RT and cryopreserved in liquid nitrogen. Analysis for cytokine release and proliferation of the cells *in vitro* was performed according to the methods described by Stebbings *et al*. 2007^57^. Briefly, 1 µg of the therapeutic mAb OKT3 (murine IgG2a, anti‐CD3, Tonbo Biosciences, San Diego, CA, USA) was wet coated in duplicates directly on 96‐well round bottom polypropylene plates (Corning). CD3/CD28 Dynabeads® (Gibco) were used as positive control and added in duplicates into the wells to give a bead:cell ratio of 1:4. Non‐treated wells were used as negative controls. For cytokine release analysis, the isolated human cells were defrosted and washed, then 2 × 10^5^ cells were added into the wells and incubated for 20 h in a humidified incubator at 37°C with 5% CO_2_. The secretion inhibitor Brefeldin A (Thermo Fisher Scientific, Paisley, UK) was added to the wells at 10 µg mL^−1^ for the last 4 h of incubation. Intracellular cytokine production was measured by flow cytometry in a FACSCanto II instrument acquiring 20000 hCD3^+^ events.

For measuring T‐cell proliferation, the isolated human cells were labelled with CellTrace™ CFSE (Thermo Fisher Scientific), according to the manufacturer’s instructions and plated at 2 × 10^5^ cells/well into the 96‐well round bottom plates prepared as mentioned above. Cells were incubated for 72 h in a humidified incubator at 37°C with 5% CO_2_. Cells were stained with specific human antibodies listed in Supplementary table 2. Activation and proliferation of T cells was measured by acquisition of 20000 hCD3^+^ cells by flow cytometry in a FACSCanto II instrument and cells phenotyped as follows: activated T cells (hCD25^+^/hCD3^+^/hCD45^+^), proliferating T cells (CFSE^Low^/hCD3^+^/hCD45^+^). Data were analysed with FCS Express Research Edition.

### OKT3 toxicity study

NSG mice were irradiated and subsequently humanised, as stated above, with either PBMCs (*n* = 6 or 7 per group), SPMCs (*n* = 8 or 9 per group) or BM‐HSCs (*n* = 5 or 6 per group) from the same adult donors (Donors 5 and 6) or with commercially obtained UCB‐HSCs cells (Donor 4) (*n* = 6 or 7 per group), in two independent studies. Additionally, irradiated NSG‐dKO mice (*n* = 8) were humanised with SPMCs from Donor 6 (Figure 6a). Human lymphocyte engraftment levels were monitored over time as described above. Only mice with a minimum of 100 hCD3^+^ cells per µL of whole blood were enrolled in the experiment before the onset of GvHD. These levels were reached at different times among the distinct humanised mouse models. At that point, blood was collected in EDTA coated tubes, and body temperature was measured using an infrared thermometer (Bokang, Wenzhou, China) before treatment. OKT3 (anti‐CD3) was subsequently administered i.v. via tail vein at a final concentration of 2 mg kg^−1^, diluted up to 100 µL with PBS. This concentration corresponds to a 1.95‐fold the dosage used in patients during the clinical trial.^9^, ^58^ Mice in the control groups were injected i.v. with 100 µL of PBS. Animals were monitored for the following 6 h to evaluate the appearance of clinical symptoms. Body temperature of mice was measured again just before experiment termination at 6h. Total blood was collected in EDTA coated tubes at sacrifice, and tissues retrieved. To determine absolute cell counts peripheral blood was directly stained with specific antibodies listed in Supplementary table 2. Erythrocytes were lysed with RBCL buffer and Absolute Counting Beads (CountBright™, Thermo Fisher Scientific) were added prior to analysis on a FACSCanto II. Single cell suspensions from the spleen of humanised mice were prepared in RPMI‐1640 medium, stained as described above and analysed on a FACSCanto II. Data were analysed with FCS Express Research Edition. Human cells were phenotyped as follows: leucocytes (hCD45^+^/mCD45.1^−^/7AAD^−^), B cells (hCD19^+^/hCD45^+^), T cells (hCD3^+^/hCD45^+^), TCR α/β complex (hTCR α_/_β^+^/hCD45^+^), CD4^+^ T cells (hCD4^+^/hCD3^+^) and CD8^+^ T cells (hCD8^+^/hCD3^+^).

### Cytokine quantification

Human cytokines in plasma were measured using the Human cytokine Th1/Th2/Th17 Cytometric Bead array kit (BD Biosciences), which includes analytes for the detection of 7 human cytokines: IL‐2, IL‐4, IL‐6, IL‐10, TNF, IFN‐γ and IL‐17A. Mouse plasma was recovered from total blood samples and stored at −80°C until used. Plasma samples were prepared following the manufacturer’s instructions and analysed on a FACSCanto II instrument.

### Statistical analysis

Littermate animals were randomly assigned to control or treatment groups, and data were analysed blinded to the group identity. GraphPad Prism 8 (GraphPad Software, San Diego, CA, USA) was used for data analysis. To assess statistical differences between two independent groups, we used the non‐paired *t*‐test. Differences before‐after in the same group were analysed with the paired *t*‐test. Comparisons between multiple datasets were assessed using a two‐way ANOVA followed by the Tukey’s multiple comparisons test. For survival analysis, Kaplan–Meier plots were represented and statistical differences calculated using the Mantel–Cox test.

## Conflicts of interest

The authors declare no conflict of interest.

## Author contributions

**Alba Matas‐Céspedes:** Conceptualization; Data curation; Formal analysis; Investigation; Methodology; Software; Validation; Visualization; Writing‐original draft; Writing‐review & editing. **Lee Brown:** Investigation; Methodology; Resources; Validation; Visualization; Writing‐review & editing. **Krishnaa T. Mahbubani:** Investigation; Methodology; Resources; Validation; Writing‐review & editing. **Bethany Bareham:** Investigation; Methodology; Resources; Validation; Writing‐review & editing. **Jackie Higgins:** Investigation; Methodology; Resources; Validation; Writing‐review & editing. **Michelle Curran:** Conceptualization; Data curation; Writing‐review & editing. **Lolke de Haan:** Conceptualization; Funding acquisition; Project administration; Supervision; Writing‐review & editing. **Jean‐Martin Lapointe:** Formal analysis; Investigation; Resources; Software; Visualization; Writing‐review & editing. **Richard Stebbings:** Conceptualization; Funding acquisition; Project administration; Resources; Supervision; Writing‐original draft; Writing‐review & editing. **Kourosh Saeb‐Parsy:** Conceptualization; Funding acquisition; Project administration; Resources; Supervision; Writing‐original draft; Writing‐review & editing.
