# Supplementary material for: Use of human splenocytes in an innovative humanised mouse model for prediction of immunotherapy‐induced cytokine release syndrome
Source: Clin Transl Immunology. 2020 Nov 4;9(11):e1202. doi: 10.1002/cti2.1202 (PMC7641894; doi:10.1002/cti2.1202)
Supplement: Supplementary file 1 [file CTI2-9-e1202-s001.pdf]

Supplementary figure 1

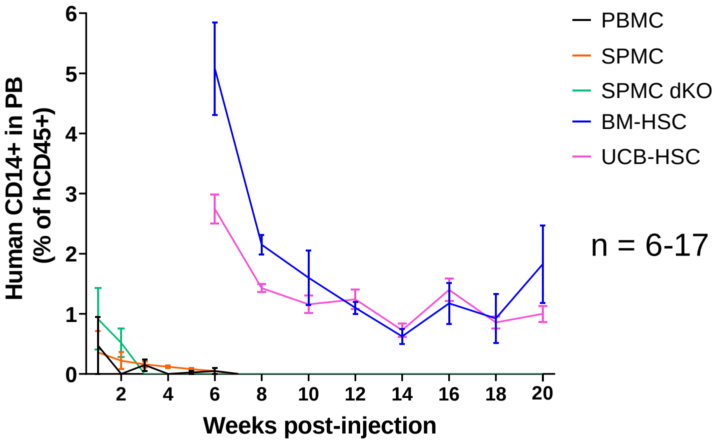

Supplementary figure 1: Distinct levels of human monocytes circulating in mouse blood in the different models.
Levels of human monocytes circulating in peripheral blood (PB) of mice, represented as a percentage within hCD45⁺ population. Mice and donor numbers are the same as in Figure 1. Mean±SEM are shown. *PBMC, peripheral blood mononuclear cells; SPMC, spleen mononuclear cells; SPMC dKO, SPMC model in NSG double knock out mice; BM-HSC, bone marrow haematopoietic stem cells; UCB-HSC, umbilical cord blood haematopoietic stem cells.*

Supplementary figure 2

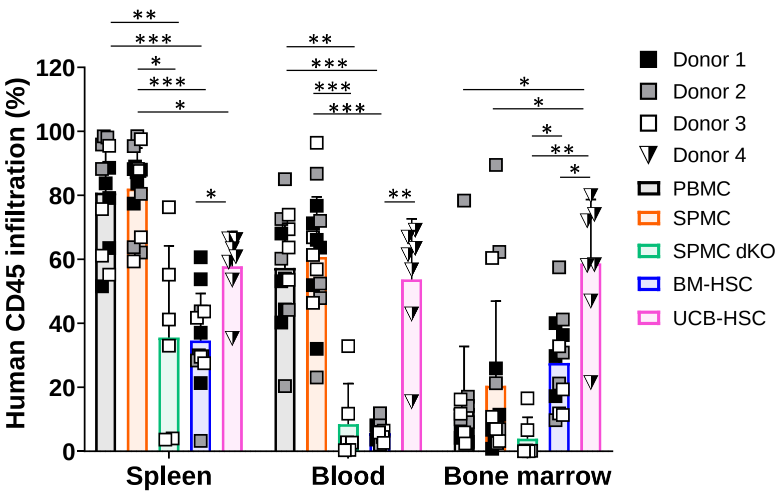

Supplementary figure 2: Human leucocyte distribution in the different mouse immune compartments.
Levels of hCD45⁺ cells in the spleen, blood and BM of the different humanised mouse models at sacrifice. Mice and donor numbers are the same as in Figure 1. Individual mouse data and mean±SD of all donors combined are shown. A two-way ANOVA was used to determine significant differences between models; *P < 0.05, **P < 0.01, ***P < 0.001. *PBMC, peripheral blood mononuclear cells; SPMC, spleen mononuclear cells; SPMC dKO, SPMC model in NSG double knock out mice; BM-HSC, bone marrow haematopoietic stem cells; UCB-HSC, umbilical cord blood haematopoietic stem cells.*

Supplementary figure 3

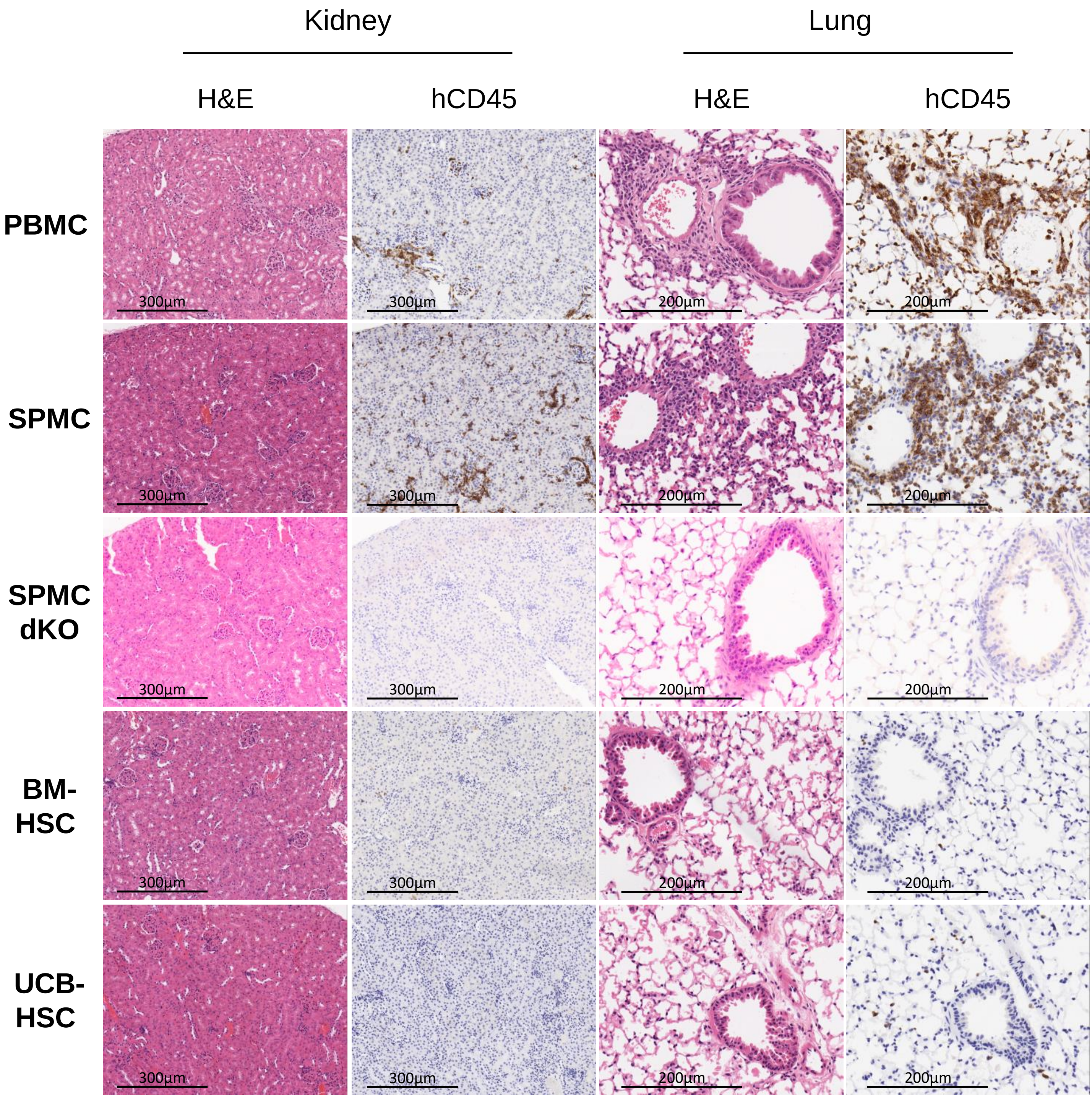

Supplementary figure 3: Histological comparison of human immune cell infiltration and inflammation levels in mouse kidney and lung.
Representative images of the H&E and hCD45 staining on the kidney and lung of the corresponding mice shown in Figure 2. Magnification: x400 for the lung and x200 for the kidney. *PBMC, peripheral blood mononuclear cells; SPMC, spleen mononuclear cells; SPMC dKO, SPMC model in NSG double knock out mice; BM-HSC, bone marrow haematopoietic stem cells; UCB-HSC, umbilical cord blood haematopoietic stem cells; H&E, haematoxylin and eosin.*

Supplementary figure 4

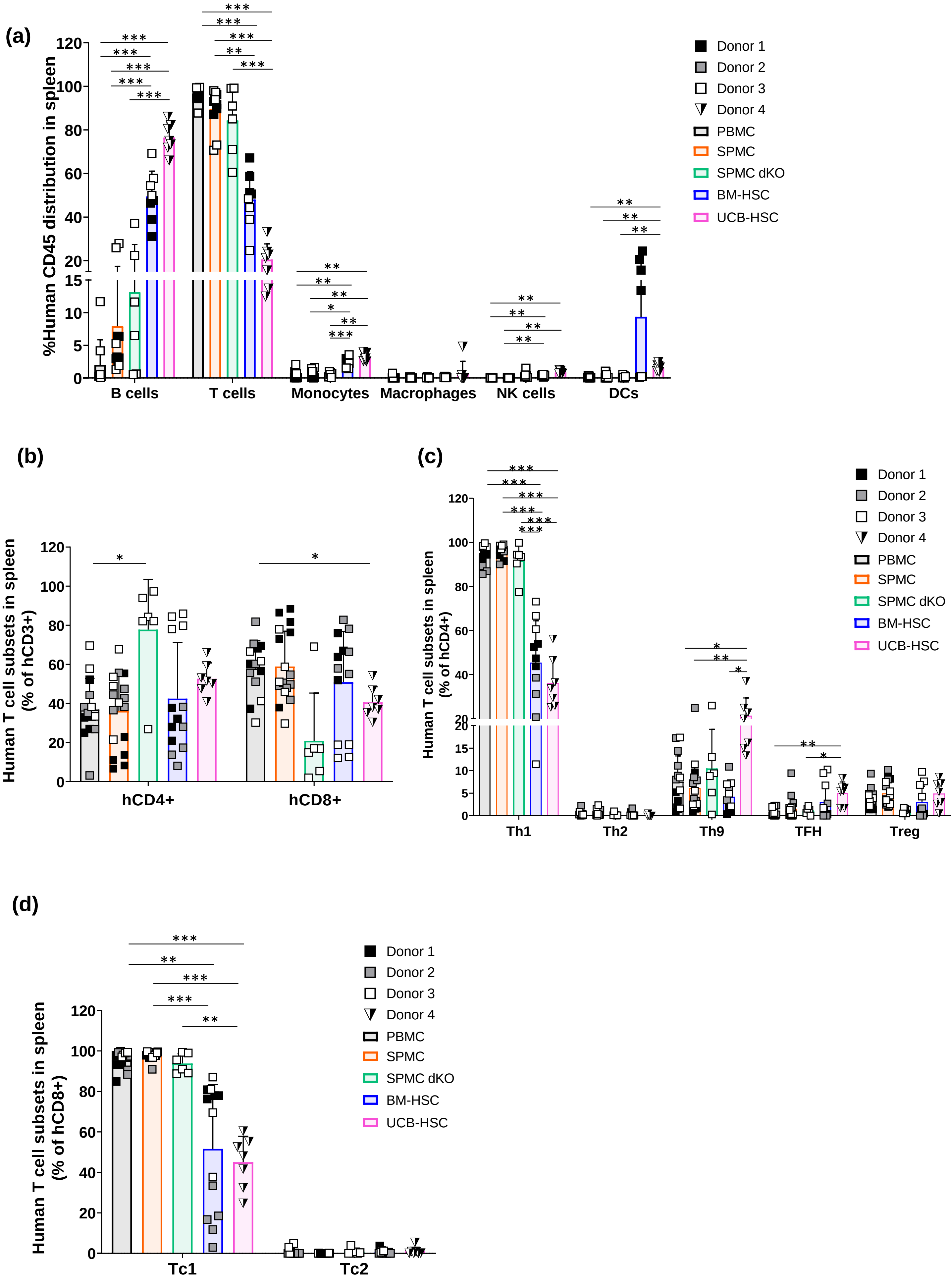

Supplementary figure 4: Subpopulations of $hCD45^+$ cells in the spleen of the different humanised mouse models.

(a) Different human immune subpopulations in mouse spleen, corresponding to the manual gating analysis done in Figure 3a. **(b)** Proportions of $hCD4^+$ and $hCD8^+$ T cells. **(c)** Percentages of T helper cell subtypes characterised within the $hCD4^+$ T cells, visually represented in Figure 3c. **(d)** Subtypes of cytotoxic T cell characterised within the $hCD8^+$ T cells. Mice and donor numbers are the same as in Figure 1. Individual mouse data and mean±SD of all donors combined are shown. A two-way ANOVA was used to determine significant differences between models; *$P < 0.05$, **$P < 0.01$, ***$P < 0.001$. *PBMC, peripheral blood mononuclear cells; SPMC, spleen mononuclear cells; SPMC dKO, SPMC model in NSG double knock out mice; BM-HSC, bone marrow haematopoietic stem cells; UCB-HSC, umbilical cord blood haematopoietic stem cells; NK, natural killer; DCs, dendritic cells.*

Supplementary figure 5

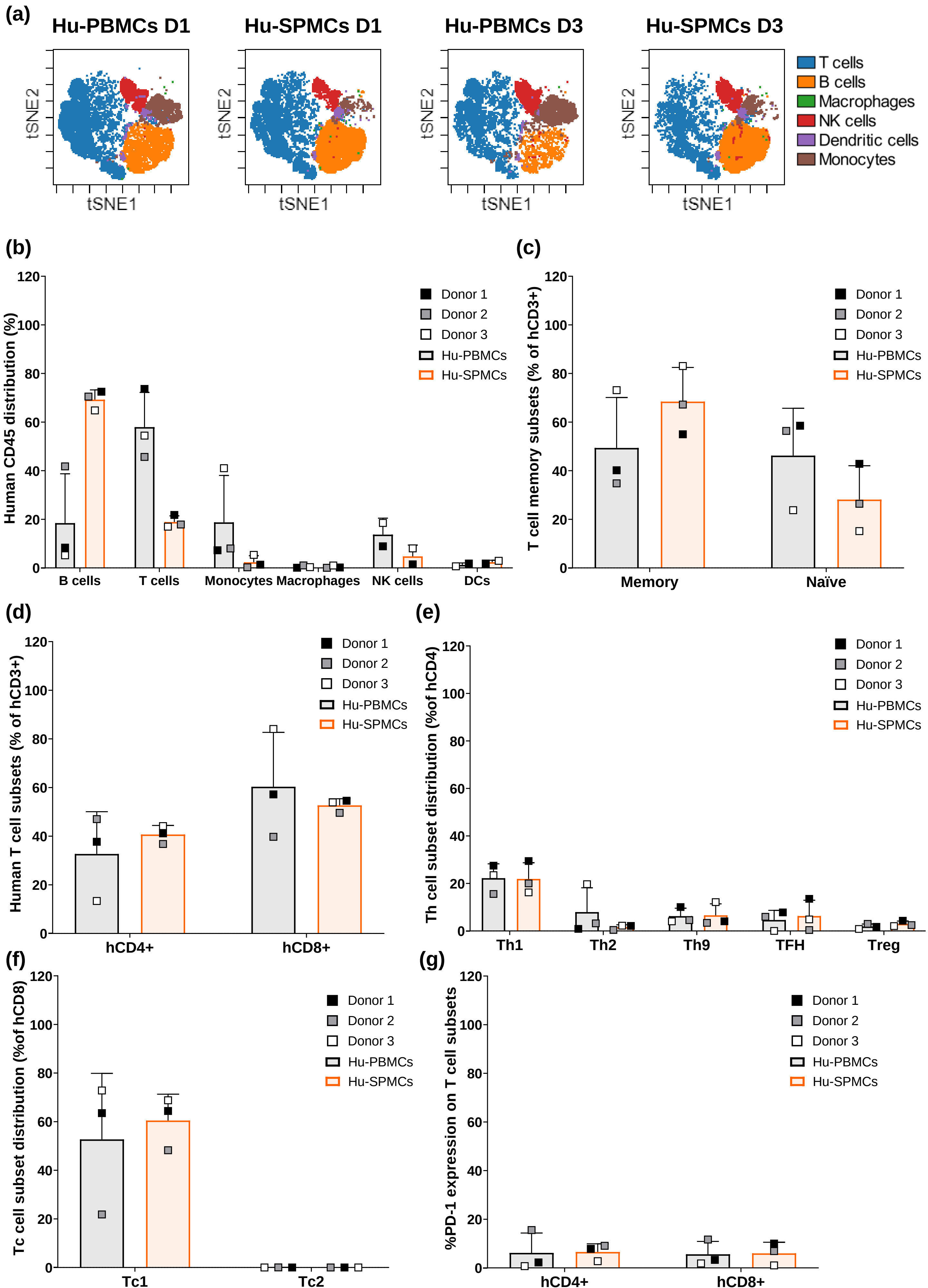

Supplementary figure 5: Immunophenotyping of hCD45$^+$ cells in human samples pre-engraftment.

(a) Visualisation using t-SNE of the immune subpopulations within hCD45$^+$ cells in hu-PBMCs and hu-SPMCs samples from Donors 1 and 3 used for engraftment in mice. **(b)** Human immune subpopulations of hCD45$^+$ cells, corresponding to the manual gating analysis done in section (a). **(c)** T cell memory phenotype determined on hCD3$^+$ cells. **(d)** Proportions of hCD4$^+$ and hCD8$^+$ T cells. **(e)** Percentages of T helper cell subtypes of hCD4$^+$ T cells. **(f)** Percentages of cytotoxic T cell subtypes of hCD8$^+$ T cells. **(g)** Expression of PD-1 evaluated on hCD4$^+$ and hCD8$^+$ cells. Data of three independent donors 1-3 are shown. Individual data and mean±SD of all donors combined are shown. Non-significant differences were found between human immune compartments (non-paired *t*-test). *Hu-PBMCs, human peripheral blood mononuclear cells inoculum; Hu-SPMCs, human spleen mononuclear cells inoculum; NK, natural killer; DCs, dendritic cells; TFH, T follicular helper.*

Supplementary figure 6

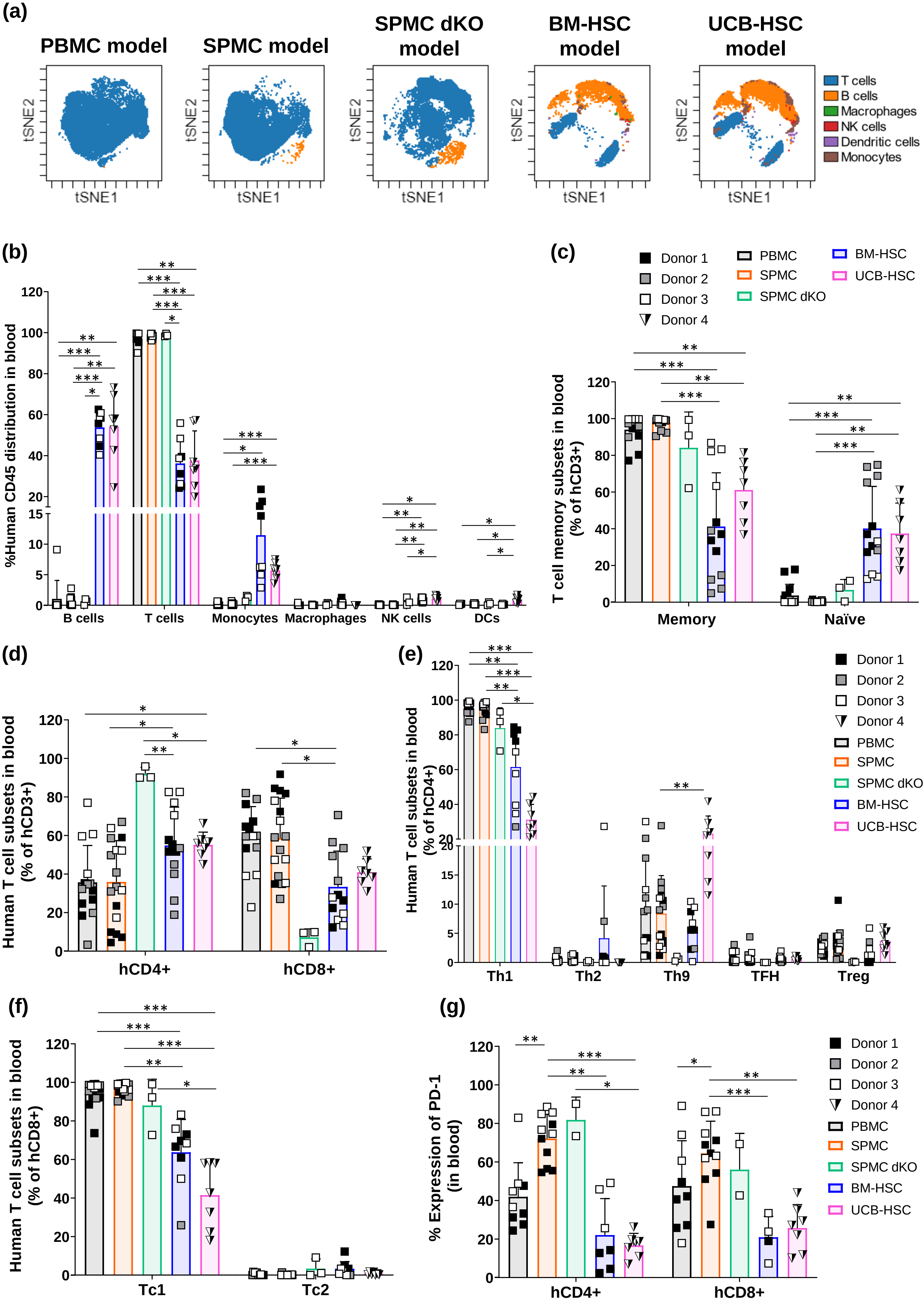

Supplementary figure 6: Subpopulations of hCD45^{+} cells in blood of the different humanised mouse models at termination.

(a) Visualisation using t-SNE of the immune subpopulations of hCD45^{+} cells circulating in mouse blood of one representative mouse in each model (Donors 3 and 4) at sacrifice. **(b)** Human immune subpopulations in mouse blood, corresponding to the manual gating analysis done in section (a). **(c)** T cell memory phenotype of hCD3^{+} T cells in mouse blood. **(d)** Proportions of hCD4^{+} and hCD8^{+} T cells. **(e)** Percentages of T helper cell subtypes of hCD4^{+} T cells. **(f)** Subtypes of cytotoxic hCD8^{+} T cells. **(g)** Expression of PD-1 evaluated on hCD4^{+} and hCD8^{+} T cells. Mice and donor numbers are the same as in Figure 1. Individual mouse data and mean±SD of all donors combined are shown. A two-way ANOVA was used to determine significant differences between models; *$P < 0.05$, **$P < 0.01$, ***$P < 0.001$. *PBMC, peripheral blood mononuclear cells; SPMC, spleen mononuclear cells; SPMC dKO, SPMC model in NSG double knock out mice; BM-HSC, bone marrow haematopoietic stem cells; UCB-HSC, umbilical cord blood haematopoietic stem cells; NK, natural killer; DCs, dendritic cells; TFH, T follicular helper.*

Supplementary figure 7

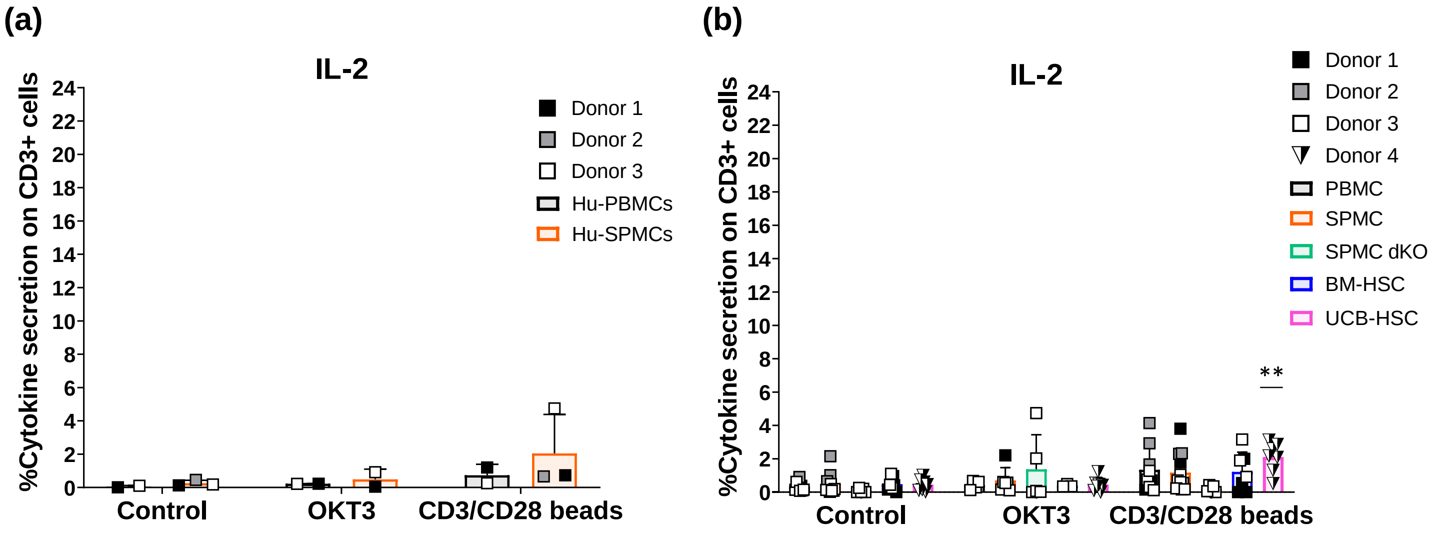

Supplementary figure 7: ***In vitro*** **production of IL-2 by T cells pre and post-engraftment in humanised mice** **(a)** Intracellular production of IL-2 on hu-PBMCs (n=2) and hu-SPMCs (n=3) samples before engraftment into mice, after 24h of culture *in vitro*. Data of three independent experiments as in Figure 4a are shown. **(b)** Intracellular production of IL-2 on human lymphocytes isolated from the spleen of humanised mice at the experiment endpoint, after 24h of culture. Mice and donor numbers are the same as in Figure 4b. Individual data and mean±SD of all donors combined are shown. A two-way ANOVA was used to determine significant differences between control and treated groups in each model; ***P* < 0.01. *PBMC, peripheral blood mononuclear cells; SPMC, spleen mononuclear cells; SPMC dKO, SPMC model in NSG double knock out mice; BM-HSC, bone marrow haematopoietic stem cells; UCB-HSC, umbilical cord blood haematopoietic stem cells; Hu-PBMCs, human PBMCs inoculum; Hu-SPMCs, human SPMCs inoculum.*

Supplementary figure 8

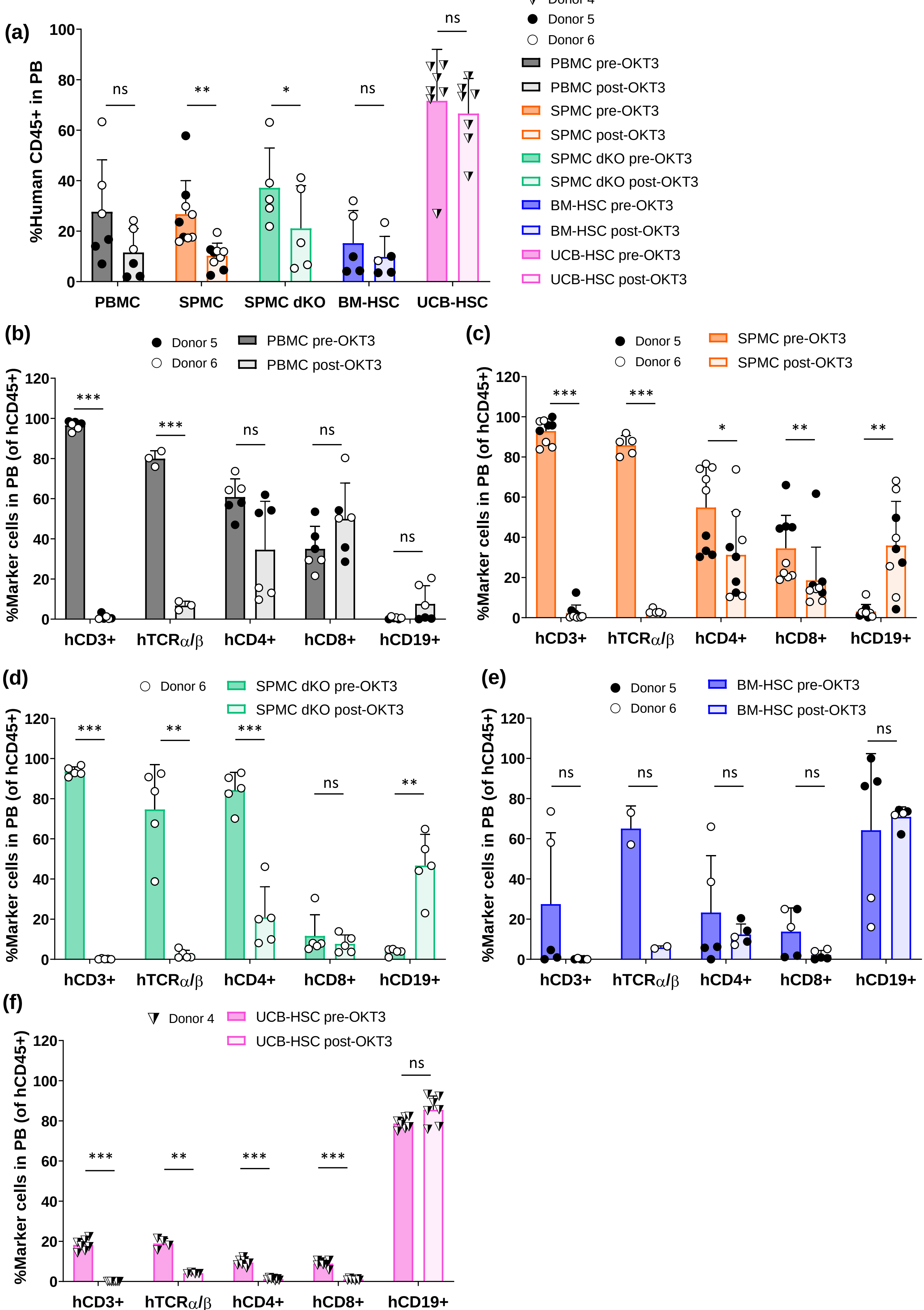

Supplementary figure 8: Decrease of hCD45$^+$ and hCD3$^+$ T cells from peripheral blood of humanised mice after OKT3 treatment

(a) Levels of hCD45$^+$ cells in peripheral blood (PB) of OKT3-treated mice, assessed before and 6h after OKT3 treatment by flow cytometry. **(b-f)** Levels of different immune cells within hCD45$^+$ population in PB, assessed before and 6h after OKT3 treatment of mice in the **(b)** PBMC model, **(c)** SPMC model, **(d)** SPMC dKO model, **(e)** BM-HSC model and **(f)** UCB-HSC model. Mice and donor numbers are the same as in Figure 6. Individual mouse data and mean±SD of all donors combined are shown. The paired *t*-test was used to determine significant differences before-after OKT3 treatment; $^*P < 0.05$, $^{**}P < 0.01$, $^{***}P < 0.001$. *PBMC, peripheral blood mononuclear cells; SPMC, spleen mononuclear cells; SPMC dKO, SPMC model in NSG double knock out mice; BM-HSC, bone marrow haematopoietic stem cells; UCB-HSC, umbilical cord blood haematopoietic stem cells.*

Supplementary figure 9

(a)

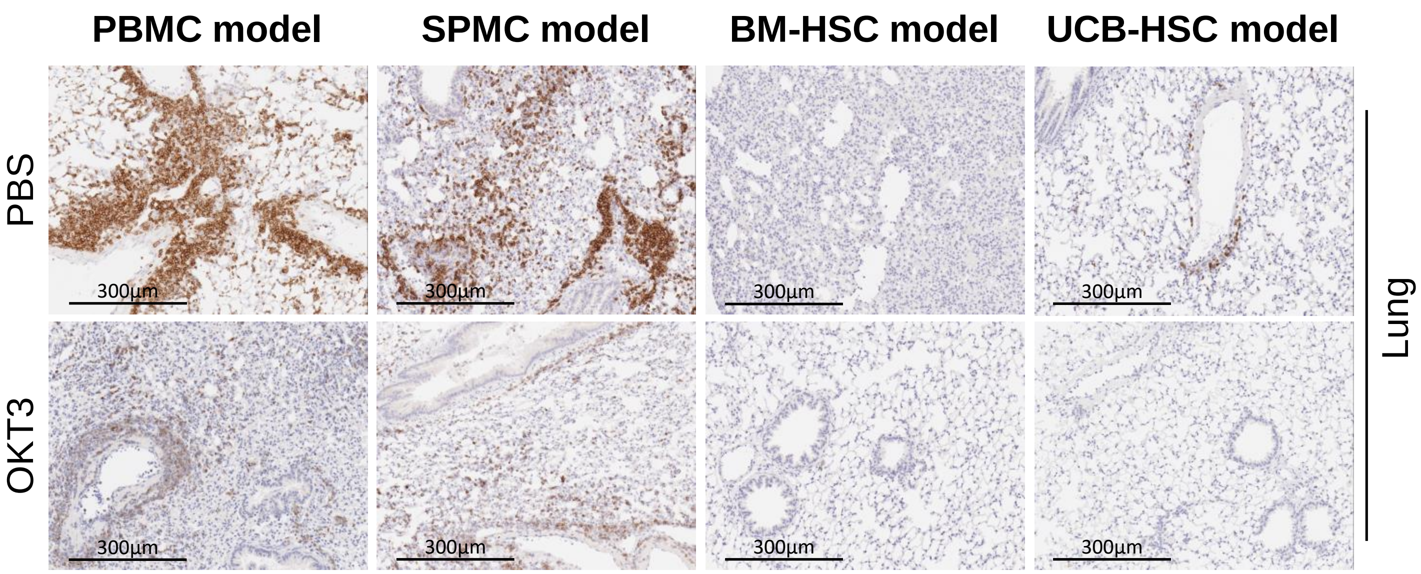

(b)

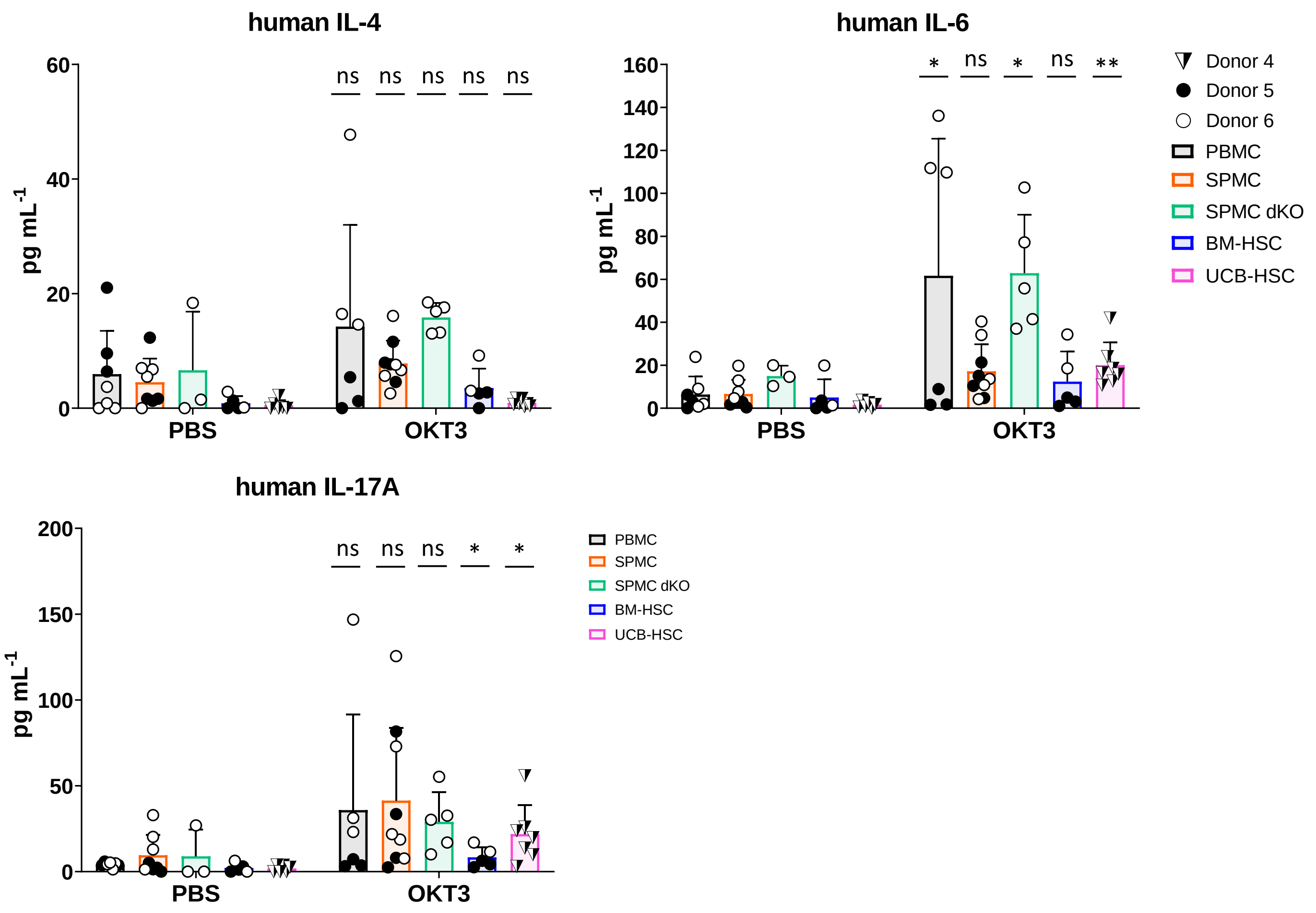

Supplementary figure 9: hCD3$^+$ T cell downregulation and cytokine release in OKT3-treated humanised mice

(a) Representative images of the hCD3$^+$ T cell staining on the lung of the corresponding mice shown in Figure 6d, magnification x200 **(b)** Human IL-4, IL-6 and IL-17A secreted into mouse plasma in PBS and OKT3 treated mice at sacrifice. Mice and donor numbers are the same as in Figure 6. Individual mouse data and mean±SD of all donors combined are shown. The non-paired *t*-test was used to determine significant differences between PBS and OKT3 treated mice in each model; *$P < 0.05$, **$P < 0.01$, ***$P < 0.001$. *PBMC, peripheral blood mononuclear cells; SPMC, spleen mononuclear cells; SPMC dKO, SPMC model in NSG double knock out mice; BM-HSC, bone marrow haematopoietic stem cells; UCB-HSC, umbilical cord blood haematopoietic stem cells.*

Supplementary figure 10

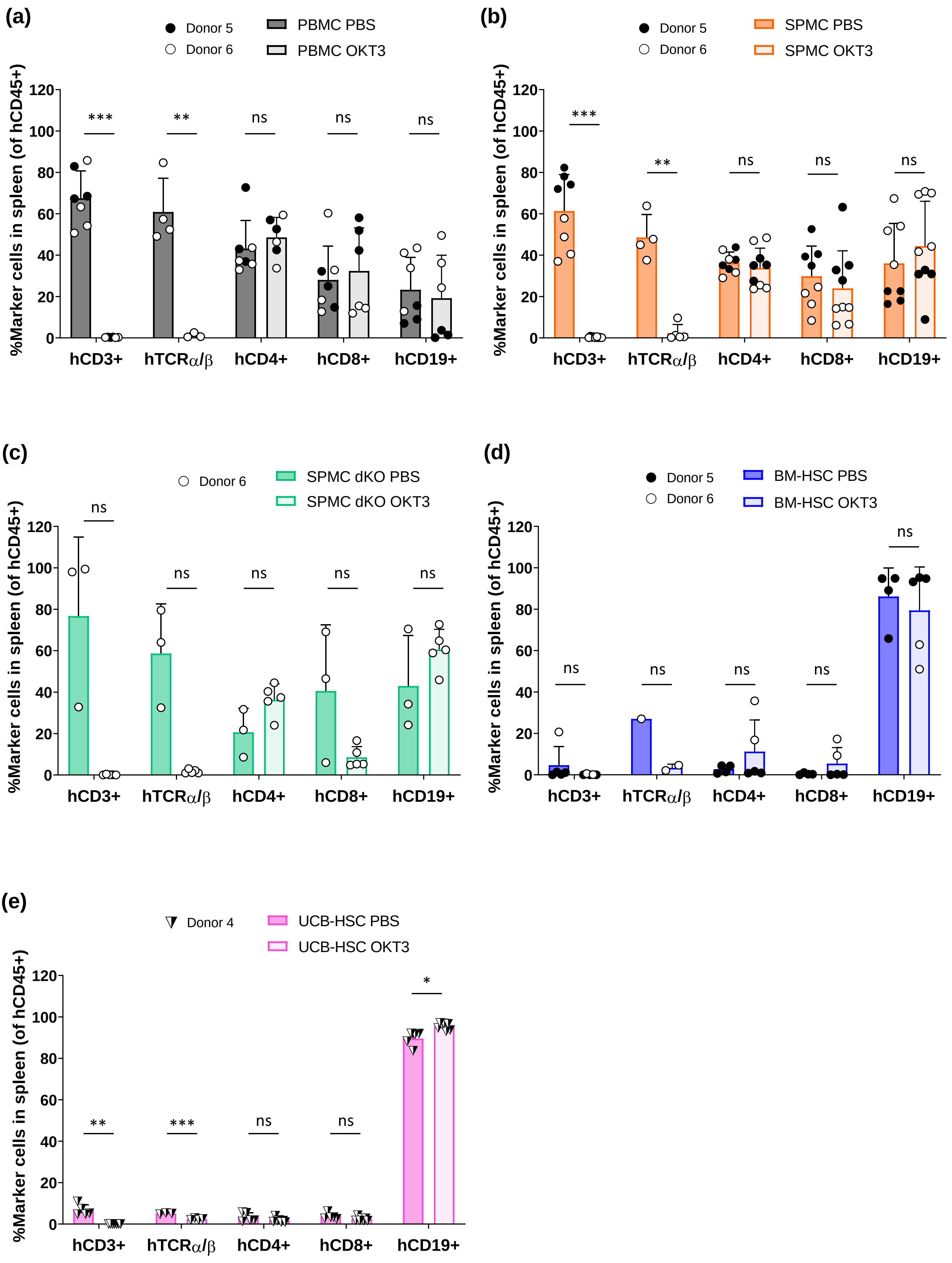

Supplementary figure 10: Downregulation of hCD3⁺ T cell expression in spleen of OKT3-treated humanised mice

(a-e) Levels of different hCD45⁺ cells infiltrating the spleen of PBS and OKT3-treated mice in Figure 6, in the **(a)** PBMC model, **(b)** SPMC model, **(c)** SPMC dKO model, **(d)** BM-HSC model and **(e)** UCB-HSC model. Mice and donor numbers are the same as in Figure 6. Individual mouse data and mean±SD of all donors combined are shown. The non-paired *t*-test was used to determine significant differences between PBS and OKT3 treated mice in each model; $^{*}P < 0.05$, $^{**}P < 0.01$, $^{***}P < 0.001$. *PBMC, peripheral blood mononuclear cells; SPMC, spleen mononuclear cells; SPMC dKO, SPMC model in NSG double knock out mice; BM-HSC, bone marrow haematopoietic stem cells; UCB-HSC, umbilical cord blood haematopoietic stem cells.*
