# Supplementary material for: Use of human splenocytes in an innovative humanised mouse model for prediction of immunotherapy‐induced cytokine release syndrome
Source: Clin Transl Immunology. 2020 Nov 4;9(11):e1202. doi: 10.1002/cti2.1202 (PMC7641894; doi:10.1002/cti2.1202)
Supplement: Supplementary file 2 [file CTI2-9-e1202-s002.docx]

| Donor | PBMC | SPMC | BM-HSC | UCB-HSC | Age | SEX | Donor type |
| --- | --- | --- | --- | --- | --- | --- | --- |
| 1 |  |  |  |  | 51 | Male | DBD |
| 2 | 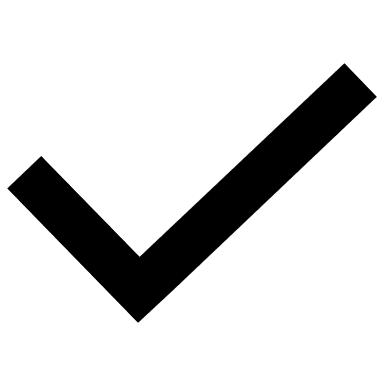 | 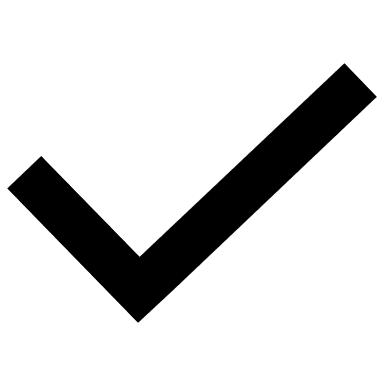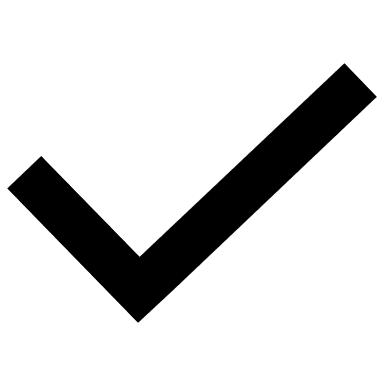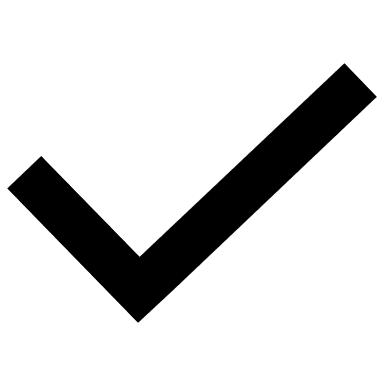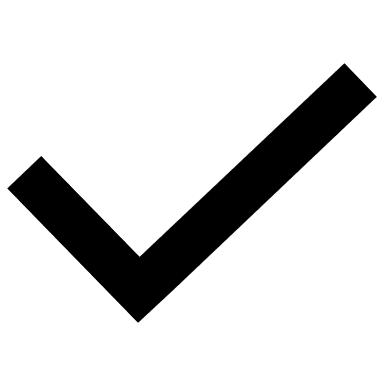 | 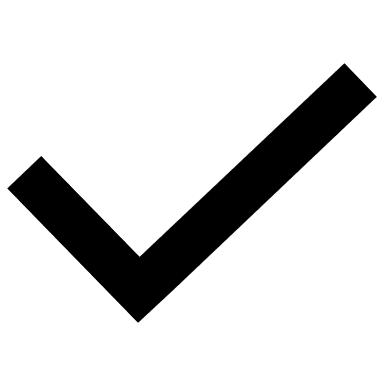 |  | 50 | Male | DBD |
| 3 | 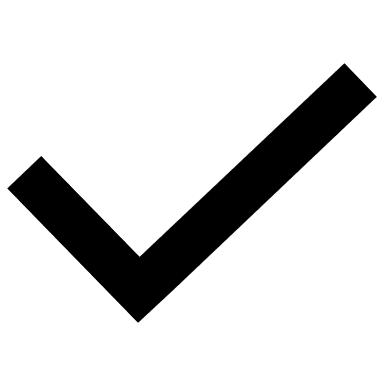 | 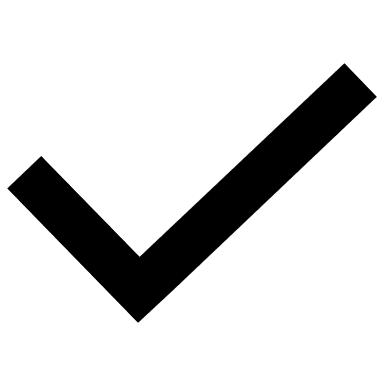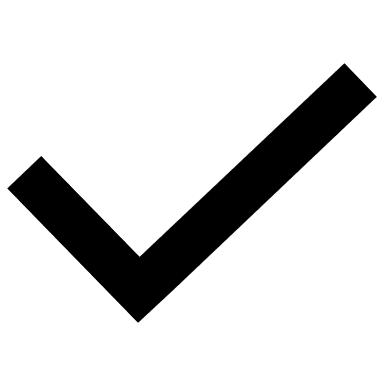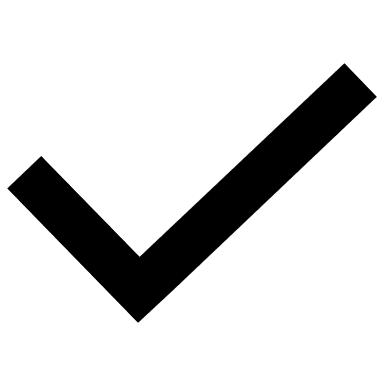 |  |  | 56 | Male | DBD |
| 4 |  |  |  | 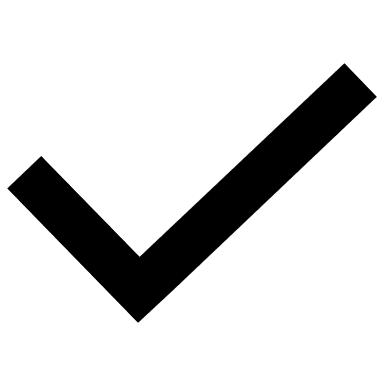 | N/A | N/A | Commercial |
| 5 | 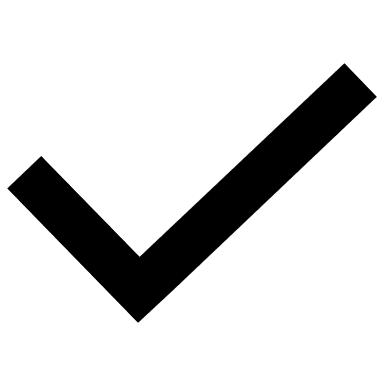 |  |  |  | 66 | Male | DBD |
| 6 | 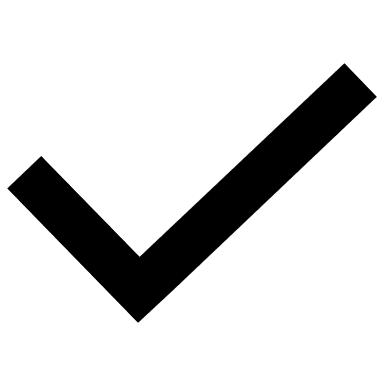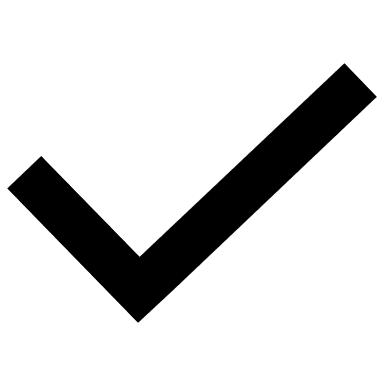 |  |  |  | 59 | Female | DCD |


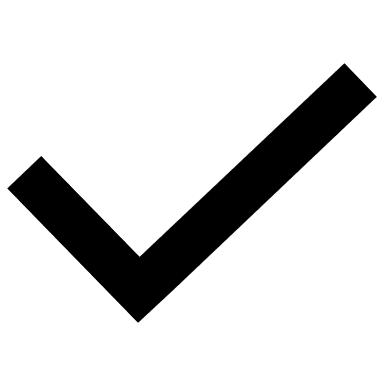

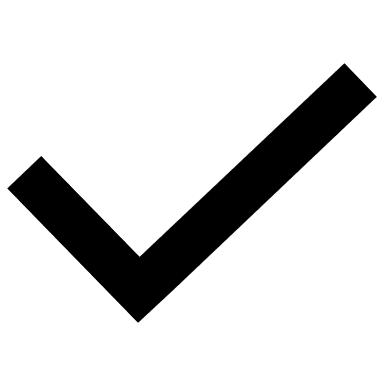


**Supplementary table 1: Donor demographics and sample availability.**

*PBMC, peripheral blood mononuclear cells; SPMC, spleen mononuclear cells; BM-HSC, bone marrow haematopoietic stem cells; UCB-HSC, umbilical cord blood haematopoietic stem cells; N/A, not available; DBD, donation after brain death; DCD, donation after circulatory death.*
