# Supplementary material for: Use of human splenocytes in an innovative humanised mouse model for prediction of immunotherapy‐induced cytokine release syndrome
Source: Clin Transl Immunology. 2020 Nov 4;9(11):e1202. doi: 10.1002/cti2.1202 (PMC7641894; doi:10.1002/cti2.1202)
Supplement: Supplementary file 3 [file CTI2-9-e1202-s003.docx]

| MARKER | CLONE | SOURCE | STAINING |
| --- | --- | --- | --- |
| hCD45 | HI30 | Thermo Fisher Scientific | Surface markers |
| hCD19 | SJ25C1 | Biolegend |  |
| hCD3 | UCHT1 | Biolegend |  |
| hCD4 | SK3 | BD biosciences |  |
| hCD8 | SK1 | Biolegend |  |
| hCD45RA | 5H9 | BD Biosciences |  |
| hCD45RO | UCHL1 | Biolegend |  |
| hCD14 | 61D3 | Thermo Fisher Scientific |  |
| hCD206 | 19.2 | Thermo Fisher Scientific |  |
| hCD11c | 3.9 | Biolegend |  |
| hCD56 | 5.1H11 | Thermo Fisher Scientific |  |
| hCD127 | HIL-7R-M21 | BD biosciences |  |
| hCD25 | BC96 | Biolegend |  |
| hCD183 (CXCR3) | G025H7 | Biolegend |  |
| hCD185 (CXCR5) | MU5UBEE | Thermo Fisher Scientific |  |
| hCD193 (CCR3) | 5E8 | Biolegend |  |
| hCD194 (CCR4) | D8SEE | Thermo Fisher Scientific |  |
| hCD195 (CCR5) | 2D7 | BD biosciences |  |
| hCD196 (CCR6) | G034E3 | Biolegend |  |
| hCD197 (CCR7) | 3D12 | BD biosciences |  |
| hCD279 (PD-1) | EH12.2H7 | Biolegend |  |
| HLA-DR | L243 | Thermo Fisher Scientific |  |
| hTCR α/β | IP26 | Biolegend |  |
| mCD45.1 | A20 | Thermo Fisher Scientific |  |
| hTNF-α | MAb11 | Thermo Fisher Scientific | Intracellular markers |
| hIFN-γ | 4S.B3 | Thermo Fisher Scientific |  |
| hIL-2 | MQ1-17H12 | Thermo Fisher Scientific |  |
| 7AAD |  | Biolegend |  |
| Fixable Viability Dye |  | Thermo Fisher Scientific |  |

**Supplementary table 2: List of antibodies for flow cytometry.**

*h, human; m, mouse.*
